# Advances in Optical Microfibers: From Fabrication to Functionalization and Sensing Applications

**DOI:** 10.3390/ma18112418

**Published:** 2025-05-22

**Authors:** Joanna Korec-Kosturek, Joanna E. Moś

**Affiliations:** Institute of Applied Physics, Military University of Technology, Gen. Kaliskiego 2 St., 00-908 Warsaw, Poland; joanna.mos@wat.edu.pl

**Keywords:** tapered optical fiber sensors, functional coatings, metallic coatings, graphene coatings, organic coatings, polymer coating, microring resonators, fiber Bragg grating, long-period grating

## Abstract

Currently, optical fibers play a leading role in telecommunications, serve as special transmission components for industrial applications, and form the basis of highly sensitive sensor elements. One of the most commonly used modifications is the reduction in the initial dimensions of the cladding and core to a few or several micrometers, allowing the evanescent wave emerging from the tapered region to interact with the surrounding environment. As a result, the microfiber formed in this way is highly sensitive to any changes in its surroundings, making it an ideal sensing element. This article primarily focuses on reviewing the latest trends in science involving various types of optical microfibers, including tapers, rings, loops, coils, and tapered fiber Bragg gratings. Additionally, it discusses the most commonly used materials for coating fiber optic elements—such as metals, oxides, polymers, organic materials, and graphene—which enhance sensitivity to specific physical factors and enable selectivity in the developed sensors.

## 1. Introduction

In recent decades, there has been a rapid increase in interest in fiber optic technology, not only in the field of telecommunications for data transmission but also in active or special transmission components for industrial applications. Fiber lasers are widely used in industries for the cutting, welding, or surface treatment of metals, and in medicine for applications such as delivering light in endoscopes, removing atherosclerotic plaque, and breaking down kidney stones. Additionally, fiber optics are utilized in a broad range of sensing technologies. This has led to the development of entirely new solutions or the enhancement of devices like gyroscopes [1], fiber lasers [2], endoscopes [3], sonars [4], hydrophones [5], and interferometers [6] by transitioning to fully fiber-based technologies.

The appropriate design of optical waveguide structures or their configurations enables the use of light to detect changes in various physical and chemical quantities. Fiber optic sensors can be categorized into several groups based on the optical beam parameters being monitored, such as amplitude, frequency, phase, or polarization, as well as the type of physical phenomena utilized, including absorption, reflection, fluorescence, etc. [7,8]. Currently, fiber optic sensors are most commonly used for monitoring changes in temperature, pressure, electric fields, magnetic fields, refractive indices (RIs), or humidity. In medicine and pharmaceuticals, they are used for detecting vitamins [9], analyzing bodily fluids such as blood or urine [10], and even performing pregnancy tests [11].

Fiber optic sensors have also found applications in industry for measuring the detonation velocity of explosives [12], monitoring crack propagation [13], and measuring stress [7], as well as for use as lighting components. The significant interest in such systems is not surprising, as fiber-based sensors are inexpensive to produce, environmentally neutral, and capable of being deployed in locations inaccessible to standard devices due to extreme conditions [14]. Their primary advantages include high accuracy and fast response times, which are incomparable to conventional sensors. Furthermore, they feature low propagation losses, do not interfere with the measured parameters, and are non-invasive. Such sensors require no complex power supply and are resistant to external electromagnetic disturbances [15].

**Figure 1:**
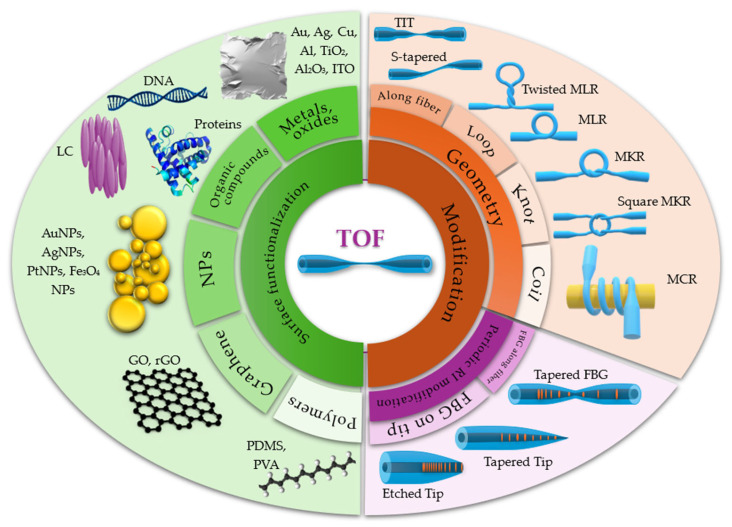
Schematic overview of common surface and structural modifications applied to tapered optical fibers (TOFs) for sensing applications. The left side illustrates surface functionalization using various materials, including metals and oxides, polymers, nanoparticles (NPs), graphene-based materials, liquid crystals (LCs), and biomolecules (e.g., DNA or proteins). The right side presents geometry-based modifications, such as loops, knots, and coils, as well as the integration of periodic structures like Bragg gratings along the fiber or on its tip.

Fiber optics used as components in devices or sensors cannot always be utilized in their original form and often require a series of modifications, such as changes to their geometry, lateral surface, or endings [16]. By sensitizing the structure in this way, it is possible to significantly improve properties and enhance the detectability of various parameters. This is achieved by optimizing sensor parameters such as sensitivity, detection range, accuracy, and resolution. Among the most common methods for increasing sensitivity are geometric modifications that use micro- or nano-sized fiber optic elements, sometimes even on the scale of a wavelength. These structures can subsequently be shaped into various geometries, such as loops or coils. Figure 1 illustrates the most common surface and structural modifications implemented on tapered optical fibers (TOFs) to enhance their sensing performance. Sensors based on TOF structures and TOF hybrid structures combined with functional materials represent a rapidly developing field, which is why they have been the subject of many reviews [17,18,19,20,21].

This paper presents an overview of the latest research on photonic sensors and devices based on optical microfibers. The first part of the article introduces the basic fabrication techniques and operational principles of microfiber-based photonic sensors and devices. The next section is divided into subsections that discuss three main types of modifications that affect sensor performance: the application of functional coatings (such as metals, graphene, polymers, and organic materials), geometry alterations (including loops, knots, and coils), and the introduction of periodic RI changes in the form of Bragg gratings. Special attention is given to the impact of various functional materials and structural modifications on the sensitivity and selectivity of the sensors. The subsequent section describes different types of microring resonators, along with an analysis of how geometry, loop size, and overlapping regions influence sensing properties. The last section is devoted to the operating principles and types of Bragg gratings, which are primarily fabricated on TOF and tapered tips. The paper concludes with a short summary of this work and a discussion on the future development prospects of TOF sensors.

## 2. Principles of Operation

### 2.1. Fabrication of the TOF

Microfibers, due to their unique optical properties and small size, serve as an excellent basis for the fabrication of more complex sensor elements. Their production methods are primarily based on the heat-and-pull technique, in which a small section of optical fiber is heated and uniformly stretched [22,23]. Several methods exist for applying heat to the TOF, including flame burners (flame brushing technique), micro-burners, lasers, filaments, or resistive heating. The flame technique can be modified, for example, by replacing the heat source with a micro-burner or a CO_2_ laser [24]. In addition to methods involving the stretching of softened fiber at elevated temperatures, there are also techniques based on the chemical [25] or mechanical removal of the cladding. Hydrofluoric acid is most commonly used for this purpose, but the etching method lacks precision, and the resulting sample is characterized by a high surface roughness, leading to significant losses.

The flame method mentioned above is the most widely used for microfiber fabrication. It typically employs a small burner powered by a propane/butane gas mixture [26], oxygen, or hydrogen [22]. The heat generated by the flame must be high enough to soften the fiber (reach the softening temperature) without causing combustion, transitioning into a liquid state, or stretching too quickly, which could introduce internal stresses and subsequent material decohesion [27]. Additionally, such setups often include computer-controlled stretching stages and laser sources with detectors to monitor losses during tapering [28]. This method enables the production of microfibers with diameters as small as tens of nanometers [28,29]. In 2014, Yu et al. described the relationship between the viscosity of silica glass and temperature [30], which helps define the fiber-forming temperature range. At lower temperatures, up to approximately 1025 °C, the high viscosity and solid state of the material result in significant internal stresses within the fiber, indicating that the process temperature is too low and must be increased. At 1120 °C, the material undergoes stress relaxation, meaning accumulated stresses are released. With further heating, the fiber begins to soften until it reaches 1670 °C, above which silica begins to melt. Based on these data, the fiber-forming temperature range is between the stress-relaxation temperature of 1120 °C and the melting temperature of 1670 °C.

**Figure 2:**
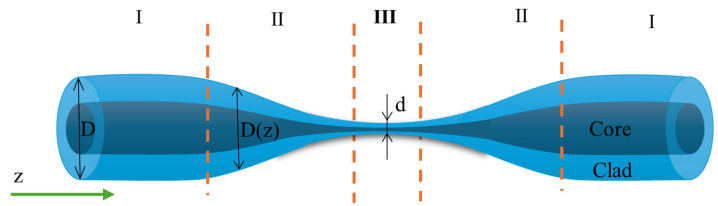
Scheme of the TOF with characteristic regions: untapered areas (I), transitions (II), and tapered waist (III).

Stretching the fiber reduces the initial fiber diameter and creates three regions: the tapered waist, transition regions, and untapered areas. Each of these sections plays a crucial role in guiding the light beam. The first transition region gradually decreases the angle of incidence of the light on the core–cladding interface, bringing it closer to the critical angle in the untapered region. In the proper taper region, light propagates throughout its volume and partially leaks out as an evanescent wave (EW). The second transition region then increases the angle back to its initial value [23,31]. Figure 2 illustrates the schematic of a TOF, highlighting its individual regions: untapered areas (I) with a diameter *D*, transition areas (II) with a varying diameter *D*(*z*) along the length *z*, and the tapered waist (III) with a diameter *d*. The length and shape of these regions can be modified by controlling the length of the heated zone and the heating duration.

Depending on the geometry and length of the waist region, TOFs can be classified into point, short, and long TOFs, whose profiles can change linearly, parabolically, or exponentially. Their characteristic shape allows for the modification of the boundary conditions of light propagating in the waist region (III); therefore, they have significant potential for sensing applications. Due to the relatively simple fabrication methods, TOFs can be made from various types of fibers. Depending on their intended application, these can include single-mode fibers (SMFs) [27], multi-mode fibers (MMFs), plastic optical fibers (POFs) [32], or photonic crystal fibers (PCFs) [33]. The high flexibility of seemingly fragile microfibers enables further shaping into structures such as microloops (MLRs) [34], coils (MCRs) [35], tips [36], couplers [37], or knots (MKRs) [34]. These structures are highly sensitive to changes in their environment and can be further enhanced through functionalization, such as doping with NPs [16] or coating with metallic oxide [38], or graphene layers [39].

**Figure 3:**
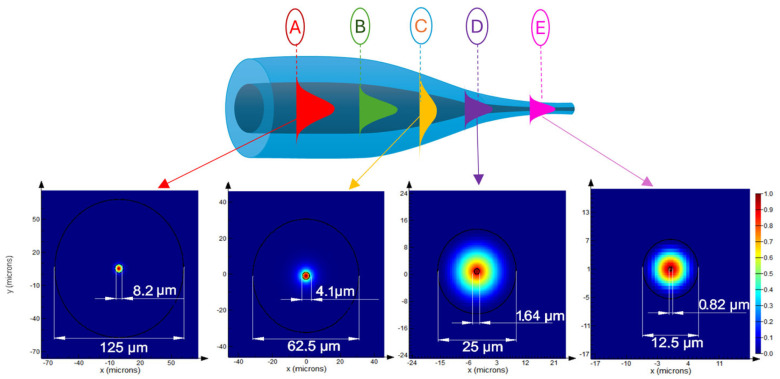
Evolution of the mode in the TOF as a function of fiber dimensions.

One of the most significant advantages of TOFs in sensing applications is the ease of optimizing their fundamental parameters, such as sensitivity, detection range, and operational wavelength range [40]. During light transmission through a waveguide with varying dimensions, the guided modes are modified in each section. As the diameter decreases, higher-order modes dissipate, leaving only the fundamental mode propagating in the TOF when a critical dimension is reached [41]. Figure 3 shows the evolution of the mode in the TOF as a function of fiber dimensions. As is well known, at point A (corresponding to standard optical fiber dimensions), the beam propagates within the core, with only a small portion penetrating into the cladding as an EW [41]. As the diameter decreases, the beam focuses within the core until reaching maximum confinement at point B. Beyond this point, the core-guided light begins to weaken, and the mode starts leaking into the cladding, eventually propagating at the cladding–air interface at point C, where the mode diameter reaches its maximum. The restriction of the mode at the cladding–air boundary causes further reductions in fiber dimensions to decrease the mode diameter, reaching a minimum at point D. For even smaller fiber dimensions, at point E, corresponding to the proper taper region, the beam becomes increasingly unbound, leaking out as an EW. This region is particularly critical for sensing applications, as it allows the guided wave to interact with the surrounding environment [41].

One of the parameters characterizing optical fibers and describing the fundamental relationship between numerical aperture, cut-off wavelength, and core radius in step-index fibers is the *V*-number (normalized frequency), which is directly related to the fiber dimensions. When the taper diameter is reduced to a specific value, d, corresponding to *V* = 2.405, only the fundamental HE_11_ mode can propagate in the TOF, or, according to the weakly guiding mode theory, the linearly polarized LP_01_ mode [42]. If *V* slightly exceeds 2.405, the fiber supports only two modes: LP_01_ and LP_11_. Careful design of the transition regions and the selection of the taper diameter can completely suppress higher-order modes.

### 2.2. EW and Sensory Properties of TOF

**Figure 4:**
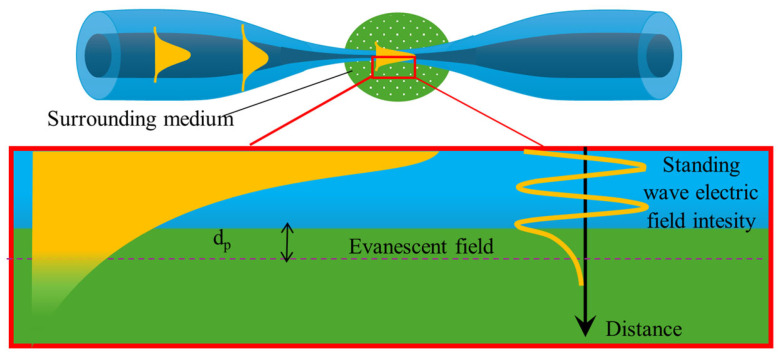
EF in TOF.

In the previous section, the evolution of mode propagation in a fiber containing a TOF was described. A characteristic feature of these elements is that as the dimensions of the fiber decrease, the beam propagating in the core begins to partially extend outside its region as an EW due to the weakening of guidance [29]. This phenomenon occurs during total internal reflection (TIR) at the interface between two dielectric media with different RIs [43]. EW is also observed within the optical fiber at the core–cladding interface [44]. However, due to the large dimensions of the cladding, only creating a TOF or removing part of the cladding allows the generated evanescent field (EF) to interact directly with the surrounding environment. This is one of the most important properties used in building optical fiber sensors, where the fiber actively participates in detecting the factors mentioned in the previous section. The TOF illustrated below in Figure 4 highlights the EW in the waist region, penetrating the medium with a lower RI and propagating parallel to the surface.

EW is a standing near-field wave whose intensity decreases exponentially with increasing distance from the interface. This phenomenon can be explained by solving Maxwell’s equations for the interface between two dielectrics. It is assumed that the tangential components of both the electric and magnetic fields are continuous along this boundary. Thus, the field at the boundary with the medium of the RI cannot abruptly drop to zero, and a small portion must penetrate the reflective medium [45]. The distance over which the electromagnetic wave penetrates the second medium is usually expressed as the penetration depth, *d_p_*. This parameter defines the perpendicular distance from the fiber–environment interface at which the electric field amplitude decreases to *1/e* of its original value. The *d_p_* value can be expressed using the following formula [44]:

(1) $$d_{p}=\frac{\lambda }{2\pi \sqrt{n_{core}^{2}\cdot \sin^{2}\theta -n_{clad}^{2}}}$$

where *λ* is the wavelength of the source, *θ* is the incidence angle of the beam at the boundary, and *n*_clad_ is the RI of the surrounding medium (e.g., the cladding in the untapered region or the environment in the tapered region).

The penetration depth into the second medium is influenced by the RI of that medium and the wavelength of light incident on the boundary. For each cladding mode, the incidence angle *θ* differs and decreases with an increase in its order. The wave reflected at the core–cladding interface experiences slight attenuation, as the lossy medium absorbs part of the optical power due to interaction with the EW [46,47]. Power absorption depends on the material’s absorption coefficient and the penetration depth of the EF into the medium. For an example structure with *n_clad_* = 1.33 and assuming *n_core_* = 1.45, the penetration depth is estimated to be approximately 140 nm for a light source with a wavelength of 470 nm [44].

## 3. Overview of Selected Sensors Based on Microfibers

Due to their characteristic structure, TOFs provide an excellent basis for constructing more complex sensors, while also serving as sensor elements themselves. They can be successfully combined with various functional materials, such as metals, oxide layers, graphene, liquid crystals (LCs), or alkanes [20,48]. Additional coatings are typically applied to enhance the sensitivity or selectivity of fiber optic sensors [21]. However, the choice of a suitable functional material for coating the taper region must meet several requirements: transparency in the designated wavelength range, the ability to influence changes in the properties of the light beam transmitted through the fiber via appropriate optical, dielectric, and material properties, rapid responses to changes in the parameter being measured, ease of integration with glass, and economic considerations in terms of cost-effective materials and inexpensive deposition/joining technology.

Furthermore, the sensitivity and selectivity of sensors based on TOFs are influenced by the RIs of the core and the surrounding medium, as well as the TOF parameters, such as length, diameter, and geometry [49,50,51]. The thickness and uniformity of the additional material layer also play crucial roles. In practice, the accuracy of measurements is also affected by factors such as the power of the light source, the detection bandwidth, and the mechanical rigidity of the sensor. Currently, there is a wide range of coating deposition techniques available, including dip coating, spin coating, layer-by-layer deposition, atomic layer deposition, electrostatic self-assembly, Langmuir–Blodgett deposition, sputtering, and chemical and physical vapor deposition [52,53,54,55,56,57,58]. It is also important to consider that creating hybrid connections between a TOF and a functional material often requires the development of appropriate protection for both the TOF and the additional material. For instance, when dealing with liquid materials, fluids, or gases, it may not always be possible to apply the material in the form of a coating or layer.

### 3.1. TOF Coated with Additional Functional Materials

#### 3.1.1. Metallic Layer Covers

The first example of combining microfibers with additional materials is their integration with metallic layers. In such a system, a wave incident from an optically denser medium (the optical fiber) and illuminating the metallic layer can lead to the generation of surface plasmons. Metals and dielectrics are characterized by dielectric constants with opposite signs, which results in the collective oscillation of electric charges at the interface of these two media, also known as surface plasma oscillation [59,60]. The quantum of these oscillations is referred to as a surface plasmon.

**Figure 5:**
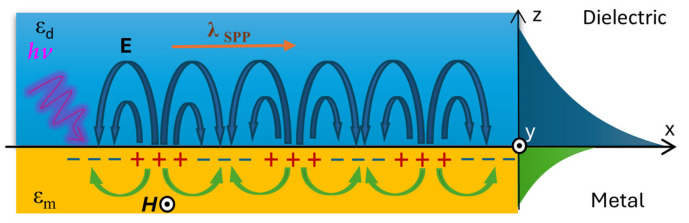
Generation of SPPs at the interface of two media: metal and dielectric.

Plasmons on the surface of the metal interact strongly with photons, forming quasiparticles known as surface plasmon polaritons (SPPs). These are achieved through the coupling of plasmons with polaritons, which are quasiparticles resulting from the interaction of an electromagnetic wave with the elementary excitations of the medium through which it propagates [60,61]. Figure 5 below illustrates the formation of SPPs at the interface of two media: metal and dielectric.

Surface plasmon polaritons (SPPs) are accompanied by the generation of longitudinal waves with TM polarization, which is why they can only be excited by waves with the same polarization. As shown in the figure, the maximum electric field is located at the metal–dielectric interface and decreases exponentially into both materials [62]. Due to the fact that the dielectric constant of metals is a complex number, the propagation constant of the plasmon, *k_SPP_*, is also complex [63]:

(2) $$k_{SPP}=k_{x}={k_{x}}^{\prime }+i{k_{x}}^{\prime \prime },$$

(3) $${k_{x}}^{\prime }=\frac{\omega }{c}\sqrt{\frac{\varepsilon_{d}\varepsilon_{m1}}{\varepsilon_{d}+\varepsilon_{m1}}},$$

(4) $${k_{x}}^{\prime \prime }=\frac{\omega }{c}\sqrt{\frac{\varepsilon_{d}{\varepsilon_{m}}^{\prime }}{\varepsilon_{d}+{\varepsilon_{m}}^{\prime }}}\frac{{\varepsilon_{m}}^{\prime \prime }}{2{\left({\varepsilon_{m}}^{\prime }\right)}^{2}}\;.$$

where *ω* is the angular frequency and *c* is the speed of light in vacuum.

The real part of the propagation constant *k_x_*′ describes the wavelength of the SPP, while the imaginary part *k_x_*″ determines the attenuation and the exponential decay of the plasmon’s electric field. This attenuation is caused by ohmic losses due to the electrons forming the SPP and the heating of the metal. It is one of the parameters that determines the spectral width of the SPP resonance peak [63]. As can be noticed from Equation (3), the properties of the surface plasmon depend on both the metal used for the coating and the dielectric. The maximum propagation constant for a wave with a frequency *ω* propagating in a dielectric can be expressed as follows:

(5) $$k_{d}=\frac{\omega }{c}\sqrt{\varepsilon_{d}}.$$

By comparing expressions (3) and (5), it follows that for the propagation constants *k_d_* and *k_SPP_* at a given frequency *ω*, the inequality *k_d_ < k_SPP_* holds. This is because the dielectric constant of metals is less than zero (*ε_m_* < 0), while for dielectrics, it is greater than zero (*ε_d_* > 0). This implies that the surface plasmon wave vector *k_SPP_* is always greater than the wave vector in the dielectric. As a result, the plasmon cannot be excited by a wave directly incident from the dielectric medium onto the interface. Instead, the EW, whose wave vector is significantly higher, can be used [59]. The wave vector of the EW in the vertical direction can be expressed as follows [64]:

(6) $$k_{ev}=\frac{\omega }{c}\sqrt{\varepsilon_{d}}sin\theta ,$$

By comparing expressions (3) and (6), the surface plasmon excitation condition can be defined as (7). As can be seen, it is fulfilled only for a specific angle of incidence of the light beam, which can be denoted as *θ_res_*:

(7) $$\frac{\omega }{c}\sqrt{\varepsilon_{d}}sin\theta_{res}=\frac{\omega }{c}\sqrt{\frac{\varepsilon_{d}\varepsilon_{m}}{\varepsilon_{d}+\varepsilon_{m}}}.$$

**Figure 6:**
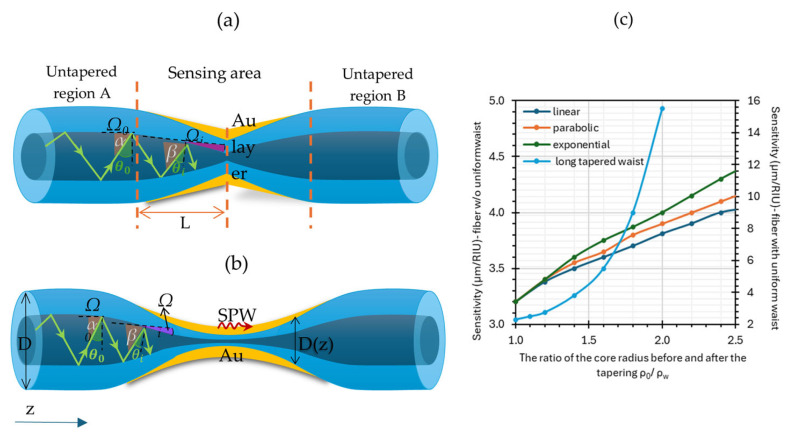
The SPR probe is based on the TOF: (**a**) without a uniform waist; (**b**) with a long and uniform waist; (**c**) sensitivity of the TOF as a function of the core taper ratio $\frac{\rho_{0}}{\rho_{w}}$.

For obvious reasons, in configurations utilizing optical fibers, it is not possible to conduct measurements as a function of the angle of incidence of the light beam. Instead, spectral measurements are performed by introducing a polychromatic light beam [65,66]. This configuration was first proposed by Villeuendas and Pelayo in 1990 [67]. Due to the phenomenon of TIR at the core–cladding interface, an EW is generated along the boundary. The coupling of the EW with plasmons is highly dependent on the wavelength, fiber parameters, and geometry, as well as the type and thickness of the metallic layer [68]. The sensitivity of an SPR sensor based on TOFs primarily depends on the shape of the taper regions: linear, parabolic, or exponential [64]. In the case of short TOFs (without a uniform long taper waist region), the angle of incidence of the beam propagating through the tapered region decreases rapidly from *θ_0_* to *θ_z_* (see Figure 6a). Additionally, due to the small core size and the reduced angle of incidence of the light beam at the core–metal boundary, the number of reflections per unit length increases compared to a prism, where it equals unity [38]. This is an undesirable effect, as the number of reflections determines the width of the resonance minimum; the greater the number of reflections, the broader the minimum becomes [64]. Meanwhile, Figure 6b shows an SPR sensor based on a TOF with a uniform tapered waist, where the angle of incidence of the beam is marked according to the position on the taper, and Figure 6c presents the sensitivity of the TOF as a function of the core taper ratio $\frac{\rho_{0}}{\rho_{w}}$ in relation to the type of geometry:

1. Short TOF without uniform waist: linear (navy blue), parabolic (orange), and exponential (green) [69];
2. Long TOF with uniform waist (light blue) [70].

Based on the figure below, it can be concluded that the reduction in the angle of incidence of the light beam is one of the most important parameters that enables an increase in sensor sensitivity (despite the increased number of beam reflections). The greatest increase is observed for a TOF with an exponential profile [70]. However, according to the literature, significantly better sensor parameters are achieved with long TOFs with uniform taper waist regions.

In the case of SPR sensors based on optical fibers, the type of metal coating significantly influences sensitivity, detection accuracy, and measurement range. As previously mentioned, metals are characterized by complex dielectric permittivity, where the real part (*ε*_r_) is responsible for reflection, and the imaginary part (*ε***_i_**) is responsible for radiation absorption [71]. The SPR spectrum is highly sensitive to both components, and therefore, the width and depth of the resonance dip primarily depend on the ratio of the real to the imaginary part *ε*_r_/*ε*_i_. The higher this ratio, the sharper and deeper the resonance minimum becomes. Table 1 presents the values of dielectric constants and the detection range of the SPR method for different metals. Based on the data in Table 1, silver and copper have the highest ratio of dielectric constants, *ε*_r_/*ε*_i_, suggesting that they are the best materials for coatings. This is indeed the case, as sensors with a silver layer exhibit the narrowest resonance minimum, while sensors with a gold layer show a much wider and shallower dip [64].

One example of an SPR sensor based on TOFs is the seawater salinity sensor developed by Wei et al. This sensor featured a TOF with a length of 27 mm, a waist diameter of 25 μm, and a 40 nm gold coating [84]. The reported sensor achieved a sensitivity of 0.708 nm/‰.

**Figure 7:**
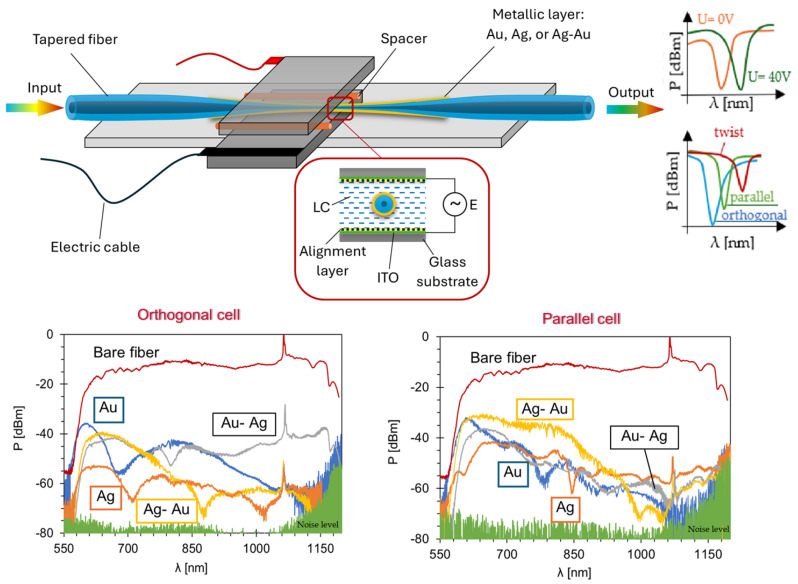
Optical fiber devices that utilize metallic coatings on TOFs and LCs.

Another example of utilizing metallic coatings on TOFs is optical devices developed at the Military University of Technology, which additionally incorporate LCs and Au, Ag, and Au-Ag bimetallic layers. Depending on the molecular alignment relative to the fiber axis—orthogonal, parallel, or twisted—and the applied voltage, a resonant dip occurs at different wavelengths (see the scheme in Figure 7) [85,86,87].

A highly effective metal for detection is palladium (Pd), which is often referred to as the “hydrogen sponge” because it absorbs gaseous hydrogen very strongly. An example of this combination is proposed by Alkhabet et al., who used a TOF elongated from an MMF with a 20 μm diameter and a 10 mm waist length [88]. The resulting sensor operates at room temperature, and its measured sensitivity, response time, and recovery time were 18,645%, 50 s, and 230 s, respectively.

Another interesting approach involves the use of TOFs in a U-shaped structure. This type of sensor was presented by a team from Guilin University in China, who utilized POFs to fabricate probes with a vertical coating of a Au film. They achieved a temperature sensor with a sensitivity of 1534.53 nm/RIU in the RI sensing range of 1.335–1.41 [32].

In 2024, a humidity sensor was constructed based on a coated polyvinyl alcohol (PVA) and Au film with a layer thickness of 40 nm. The obtained humidity sensitivity was 1.542 nm/%RH in the range of 46 %RH to 93 %RH, while the sensitivity to a temperature of −0.152 nm/°C was between 20 °C and 50 °C [89]. In 2025, a biosensor for detecting bovine serum albumin (BSA) was proposed [90].

In contrast to SPR sensors, which utilize continuous metallic layers, there are sensors that employ metal NPs of various shapes and sizes. In such elements, the localized surface plasmon resonance (LSPR) effect is observed. The main advantages of using NPs include lower production costs, the ability to create smaller sensor elements with high spatial resolution, and improved sensitivity. It can be particularly beneficial for detecting small particles, which is why such sensors are mainly used as biosensors [16]. An example of such a connection could be a sensor based on a multilayer consisting of Al_2_O_3_-Ag-Au deposited on a TOF elongated from an MMF, with AuNPs added for higher sensitivity [90]. The sensor operates within a BSA concentration range of 1 ng/mL to 10 mg/mL, with a high sensitivity of 19.46 nm/log(ng/mL) and a low limit of detection (LOD) of 0.3 ng/mL.

An interesting approach is the use of the hollow AuNPs, characterized by a thickness of 2.5 μm and a diameter of 50 nm, along with a TOF with a 10 μm diameter, which were proposed as RI sensors to detect cancer cells by Borkijkhani [91]. The hollow AuNPs exhibit better sensitivity and stronger resonance compared to solid AuNPs.

The literature also includes sensors based on iron NPs (FeNPs). Kharissova et al. proposed a sensor based on Alizarin Red S, utilizing two TOFs separated by 10 mm, with a taper waist diameter of 40 μm [92]. The fibers were coated with a layer of multi-walled carbon nanotubes functionalized with groups such as –COOH or –OH, as well as multi-walled carbon nanotubes with FeNPs. There are many similar examples of biological and humidity sensors based on TOFs and metals or NP layers.

Metal layers and NPs are attractive materials for coating TOF sensors due to their ability to enable detection via SPR or LSPR phenomena. Future perspectives focus on using metals at the nanoscale—such as NPs—combined with other materials to improve the performance parameters of the constructed devices. Recent articles describe such hybrid combinations, and this trend is expected to expand in future research.

#### 3.1.2. Oxide Coatings

In addition to metallic layers, fiber optic sensors may also contain coatings of metal oxides or other double-layer materials. It has been proven that materials with a high RI, such as titanium dioxide (TiO_2_), zinc oxide (ZnO), indium tin oxide (ITO), indium(III) oxide (In_2_O_3_), tin oxide (SnO_2_), indium oxide (In_2_O_3_), and aluminum oxide (Al_2_O_3_), improve sensitivity and accuracy, while ensuring long-term stability [38,93,94]. These materials have mainly been applied as sensors for detecting gases and changes in RI and have later been used in biochemical sensors as well.

An interesting phenomenon that occurs when optical fibers are combined with certain oxides or polymers is lossy mode resonance (LMR) [57]. Sensors based on this phenomenon are coated with a layer characterized by a high absorption coefficient, where the real part of the dielectric permittivity is positive and higher in magnitude than its imaginary part. Additionally, it must be higher than the real part of the material surrounding this layer. Light guided in the TOF in the form of EW couples with a lossy mode formed in the extra layer [95]. One of the most important parameters, aside from the permeability value, is the layer thickness. As the film thickness increases, more lossy modes are guided by an extra layer [95,96].

Equation (8), which helps to understand the phenomena of LMR, is described by the following [96]:

(8) $$n_{eff}=n_{p}\sin\theta_{i}.$$

**Figure 8:**
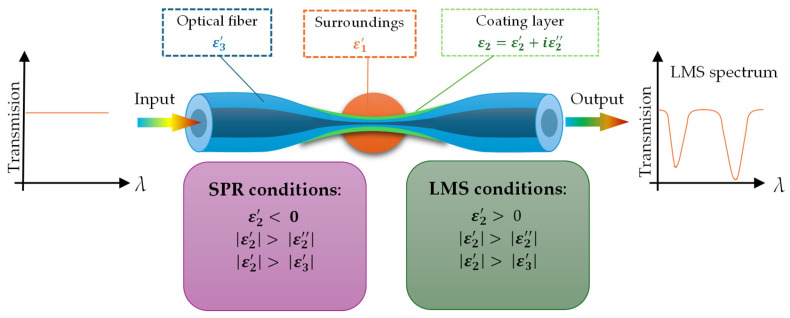
Scheme and conditions of generation of LMS.

In Equation (8), *n_eff_* is the effective RI of the EW, *n_p_* is the RI of the substrate, and $\theta_{i}$ is the incident angle of the light. The LMS phenomenon is observed when the effective index of the EW matches the effective index of lossy modes at a particular angle or wavelength. The scheme of the LMR-based sensor is presented in Figure 8.

If we compare SPR and LMS effects, the main differences are as follows [97]:

- The permittivity value of the coating layer is higher than 0 (opposite to the SPR effect);
- The number of attenuation dips in LMR of more than one can be observed on the spectrum;
- LMS phenomena can be observed in a wide wavelength range—VIS and NIR;
- In the case of LMS, as the layer thickness increases, the number of dips and the wavelength range at which the effect can be observed increase;
- In comparison to SPR, where a film of metal is required, for LMS, a different material can be used, like polymers, oxides, or combinations of polymers and NPs;
- TM- and TE-polarized light can generate this effect in sensors.

Examples of oxides where this phenomenon can be observed include TiO_2_, ITO, ZnO, Al_2_O_3_, In_2_O_3_, and SnO_2_. Moreover, coatings of ITO and ZnO fulfill the conditions for both SPR and LMS effects [97]. Optical fiber sensing structures like D-shaped fibers, unclad optical fibers, TOFs, and taper-in-taper (TIT) structures with oxide layers were used. Table 2 presents the values of dielectric permittivity for different oxides and silicon dioxide (SiO_2_) as a reference.

One example, developed by Ascrobe et al. [101], is a sensor operating on the principle of LMR, consisting of a unilaterally coated TOF with a layer of ITO. In sensors utilizing an ITO layer, devices can work on the principle of SPR and LMR depending on the operating wavelength. These authors developed a humidity sensor based on a TOF with a diameter of approximately 35 μm [101]. 

Tiwari et al. [102] demonstrated an ammonia sensor based on a TOF coated with a TiO_2_ layer operating on the LMR principle. The sensor exhibited a sensitivity to concentration changes of 1 ppm and a response time of under 1 min.

Vicas et al. [103] analyzed the influence of taper profiles, taper ratios, and ITO/AZO (aluminum-doped zinc oxide) coatings on sensor sensitivity. For the ITO layer, the optimal configuration was an exponential–linear taper profile, with sensitivity values of 12.005 μm/RIU and 0.8 μm/RIU for the first and second lossy modes, respectively, at the taper ratio of 1.7. For AZO, the corresponding sensitivity values were 0.515 μm/RIU and 0.235 μm/RIU at a taper ratio of 2.0. In the future, this configuration may be applied in chemical and biological sensing.

Zhu et al. developed a device based on an adiabatic TOF and a TiO_2_ oxide layer. A refractometer created by depositing TiO_2_ with a thickness of 50.9 nm on the TOF showed an RI sensitivity of up to 7096 nm/RIU in the SRI range of 1.3373–1.3500. Due to the advantages of TiO_2_, such as its high RI, non-toxicity, and good biocompatibility, this refractometer is expected to have wide applications in the field of biochemical sensors.

Another oxide applied by the same authors for coating adiabatic TOFs is Al_2_O_3_ [38]. This sensor demonstrated a high sensitivity of 60,008 nm/RIU. Yahya et al. developed a hydrogen (H_2_) sensor based on a TOF coated with a manganese dioxide (MnO_2_) nanostructure and a Pd layer deposited to enhance H_2_ detection [104]. Wang and colleagues proposed a sensor based on a SnO_2_ thin film and SnO_2_ NPs, operating on the LMR principle [105]. The sensor demonstrated a remarkable sensitivity of up to 5334 nm/RIU in theoretical analysis and 4704 nm/RIU in experimental conditions [105].

**Figure 9:**
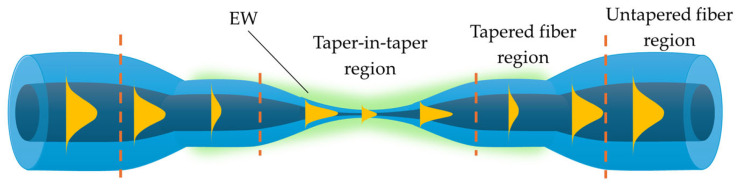
Scheme of TIT structure.

Similar to metallic layers, NPs of oxides are also used in TOF-based sensors. Liu proposed a new structure called a TIT connected with ZnO-NPs. The process of fabrication contains two stages: an elongation of a traditional TOF and a subsequent second elongation of the waist region of the first TOF [106]. Figure 9 presents the scheme of the TIT sensor. The process enhanced the sensitivity of the structure for changes in the RI, whereas the ZnO-NPs were used to detect creatine with a high sensitivity of 0.11 a.u./μM for creatinine solutions.

Moreover, the hybrid composite of AuNPs/MoS_2_-NPs (molybdenum disulfide nanoparticles) and CeO_2_-NPs (cerium oxide nanoparticles) was used on sensing areas in TIT structures to detect alanine aminotransferase. The working principle of this sensor was LSPR due to AuNPs. In this case, the MoS_2_-NPs and CeO_2_-NPs provided biocompatibility and stability for the layer. The sensitivity is 4.1 pm/(U/L) over a linear range of 10 to 1000 U/L [107]. Another example is the use of S-tapered fiber and SiO_2_ NPs to build a humidity sensor. Sensitivities of 1.1718 nm/%RH and 0.441 dB/%RH have been achieved for a high humidity range of 83.8%RH to 95.2%RH [108]. Many such examples of sensors can be found in the literature.

Future developments should focus on hybrid coating solutions, where oxides are used as additional materials in the form of NPs. The examples mentioned above support this assumption and provide directions for future research.

#### 3.1.3. Organic Compound Coatings

Organic compounds are frequently used as a cover of TOF in various configurations. There is a vast selection of these materials depending on their functional groups and other properties.

One commonly selected organic compound for use with optical fibers is porphyrin, which strongly absorbs electromagnetic radiation and exhibits intense fluorescence under UV light. For example, a coating of tetrakis-(4-sulfonylphenyl) porphyrin and poly(allylamine hydrochloride) was applied to a TOF elongated from an SMF with a diameter of 10 μm and used as an ammonia (NH_3_) sensor [109]. Another team, led by Tiwari, developed an NH_3_ sensor using a coating of TiO_2_ and porphyrin [110].

An interesting material connected with a TOF is the zeolite imidazole framework ZIF-8. It is a class of metal–organic frameworks. It is usually used in gas sensing because the material is characterized by high porosity and thermal and chemical stability. Wang et al. proposed an ethanol gas sensor. The sensor works based on the Vernier effect. The sensitivity was about 18,366.17 nm/RIU [111]. Moreover, the TOF region was functionalized by coating it with aldehyde modifiers to detect Leptospira DNA. The research was presented by Zainuddin et al. in 2018 [112]. The sensitivity of the sensor was 1.2862 nm/nM, and it was able to detect as low as 0.1 fM [112].

**Figure 10:**
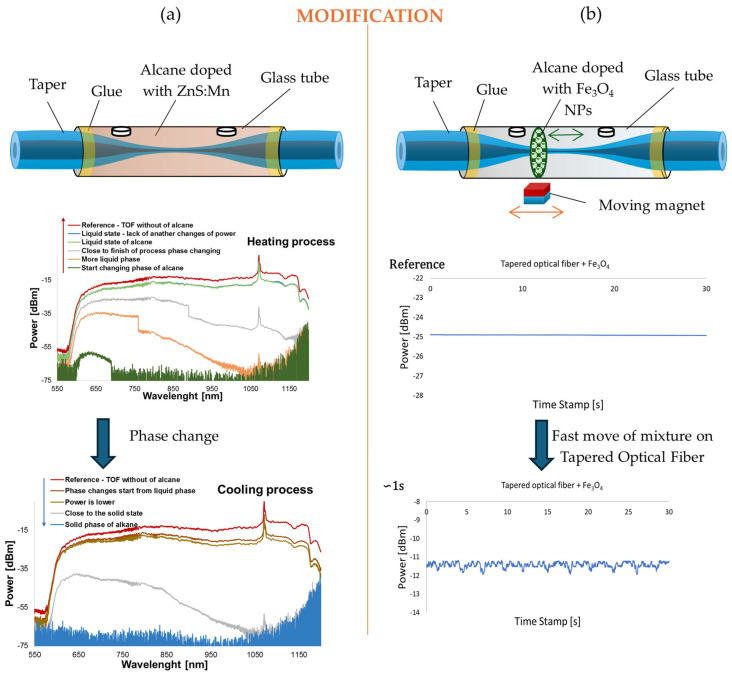
The TOF immersed in alkane doped with NPs: (**a**) alkane + NPs of ZnS:Mn, forming an ON–OFF optical switcher, and (**b**) alkane + NPs of Fe_3_O_4_, forming a movable liquid controlled by an external magnetic field, were used to build polarization switches.

Another example of using organic materials as an external medium for a TOF is the application of higher alkanes, such as hexadecane (C_16_H_34_) and heptadecane (C_17_H_36_), doped with zinc sulfide NPs doped with manganese (ZnS:Mn NPs) (see Figure 10a). Depending on the ambient temperature, these materials can exist either as a solid or a liquid. As a result, variations in transmitted power during the phase transition enable the development of a switcher or a sensor operating in an ON–OFF mode. Initial transmission measurements with pure alkanes exhibited significant hysteresis between ON and OFF states. However, the addition of just 1–5% ZnS:Mn NPs significantly reduced this effect by improving thermal conductivity and introducing extra nucleating agents [113].

Higher alkanes can also be doped with magnetic NPs, such as iron (III) oxide NPs (Fe_3_O_4_ NPs). The resulting mixture, containing 2 wt.% of dopants, remains in a liquid state at room temperature and can be controlled by external factors such as a magnetic field. Such studies were conducted by Stasiewicz et al., demonstrating that depending on the placement of the mixture along different sections of the TOF, changes occur in transmission properties (power and loss) as well as polarization characteristics, including azimuth and ellipticity. A schematic representation of the experiment is shown in Figure 10b [114].

Liquid crystals (LCs) can also be used as active surroundings of TOF. These materials provide possibilities for changes in the RI by steering molecules with magnetic or electric fields and temperature. TOFs detect these. Not all mixtures of LCs can be used with optical fibers. The most important parameter is ordinary and extraordinary RIs and the averaged effective RI, which “see” the optical fiber. Usually, if the RI of the LC is higher than the RI of the fiber, then light leaks out or losses are high. The most popular LCs applied with optical fibers are different types of mixtures of 1550, 6CHBT, 3092, E7, etc. [115]. Niewczas et al. built special cells that enable a steering molecule of LCs around the TOF. TOFs produced on standard SMF and 6CHBT were used [116]. The diameter of the TOF was about 15 μm with a low loss of about 0.5dB@1550nm. In addition, the LC was doped with magnetic Fe_3_O_4_NPs with concentrations of 0.1 wt.% and 0.5 wt.%. Doping of NPs decreases in switching times. Another work presents research based on TOFs and a mixture of LC 1550*-doped AuNPs with varying concentrations of 0.1 wt.% and 0.3 wt.%. Doping of 0.1 wt.% improves the parameters of the LC cell, but 0.3 wt.% was characterized by high losses due to aggregates in the middle of the cells [117].

#### 3.1.4. Polymeric Coatings

Another functional material used as a coating for TOFs is organic compounds belonging to the polymer group [118]. These polymers find applications as chemical sensors for detecting gases, biochemical sensors, humidity sensors [119], pressure sensors [120], and more. Some of them also generate the previously described phenomenon of LMR [121]. Soccorro et al. [121], as a first publication, conducted theoretical analyses and laboratory studies on a TOF elongated from an SMF combined with polymers such as PAH, PAA, or poly(styrene sulfonate) (PSS), employing the LMR phenomenon. Simulations demonstrated that using an SMF TOF with a polymer layer where LMR was generated could successfully detect anti-gliadin antibodies (AGAs) at a concentration of 5 ppm. This application could be used for diagnosing gluten intolerance and shows the potential of this technology in biosensor applications.

Another example is a paper moisture content sensor based on TOFs elongated from POFs coated with polyvinyl alcohol (PVA) and diatomaceous earth (DE).-. The sensor sensitivity of 0.1662%/% ω was achieved. The thickness of the layer was 90 μm, which was deposited by a layer-by-layer deposition method [122].

**Figure 11:**
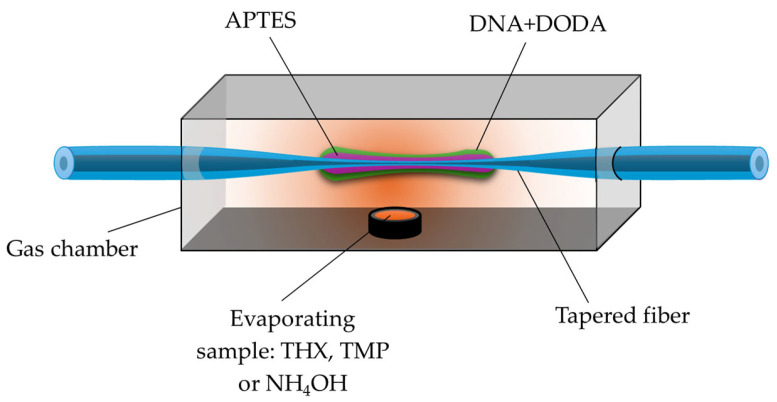
Experimental scheme using a TOF coated with a DNA biopolymer.

In the study by Stasiewicz et al., a sensor for volatile liquid vapors—THX, TMP, and NH_4_OH (imitating hazardous agents)—was developed based on a TOF coated with a biopolymer layer of deoxyribonucleic acid (DNA) combined with the cationic surfactant DODA (see Figure 11). The presented sensor was tested across a broad spectral range of 600–1200 nm, where power variations were observed during exposure to the selected vapors [123].

Wang et al., in 2024 [124], presented a sensor with bending directions and a body temperature based on TOFs and a hybrid connection of two materials: MoS_2_ (molybdenum disulfide) and PDMS (polydimethylsiloxane). The combination of these materials provided a higher sensitivity of the sensor. The sensitivity response was on the level of 23.62 nm/m^−1^ in reverse bending and 2.70 nm/m^−1^ in obverse bending at a curvature range of 4.31 to 6.10 m^−1^. The sensitivity is 1.10 nm/°C in the temperature range of 35 to 42 °C [124].

The PDMS layer on TOF is also used in [125] as a temperature sensor with a sensitivity of 76.8 p.m./°C. The configuration of the sensor was a Mach–Zehnder interferometer (MZI). Dai et al. [126] proposed a biosensor based on a TOF and a PDMS with a surface-modified nanohemisphere array. PDMS is often used due to its high flexibility. The array has a role in improving sensitivity and eliminating misalignment. In summary, the sensor was characterized by a sensitivity of 0.0138 mV/kPa, with a rise time of 3.7 ms and a fall time of 6.26 ms. A subsequent curvature sensor based on a TOF with a polyester polymer was proposed. The advantage of the sensor is its independence of temperature in the range of 24–90 °C and sensitivities of −5.017 dB/m^−1^ and 5.862 dB/m^−1^ in the curvature ranges from 1.23 to 4.44 m^−1^ and from 4.64 to 7.54 m^−1^ [127].

One interesting solution is the use of an innovative TIT structure as the foundation for a sensor detecting Cu^2+^ ions in drinking water. In 2022, Gong et al. proposed a highly sensitive sensor incorporating a TIT covered with a polyelectrolyte layer (chitosan/polyacrylic acid film) fabricated using mode–mode interference. The sensor exhibited a sensitivity of 78.03 nm/mM for Cu^2+^ concentrations ranging from 0 to 0.1 mM, while for concentrations between 0.1 and 0.7 mM, the sensitivity was 14.59 nm/mM [128].

Moreover, the PDA (polydopamine)/AuNPs as a hybrid connection was applied to the TOF structure. The principle of working this sensor was an EW. The gluconic acid aqueous solution was monitored. Wang et al. concluded that the use of additional material resulted in an improvement in the sensitivity of 80.7% in comparison to bare optical fibers [129]. Another TOF structure with a PVA coating for humidity measurements was proposed by Chen et al. in 2020 [130]. The humidity sensitivity was 0.1194 nm/%RH. The advantage of the sensor was a low-temperature sensitivity of 0.029 nm/°C [130]. 

Polymeric compounds, due to their flexibility and biocompatibility, are being increasingly used. Recent research indicates a growing trend in the use of a hybrid integration of applied materials to enhance the sensitivity and selectivity of sensors, as well as to improve their stability.

#### 3.1.5. Graphene, Graphene Oxide, and Reduced Graphene Oxide Coatings

**Figure 12:**
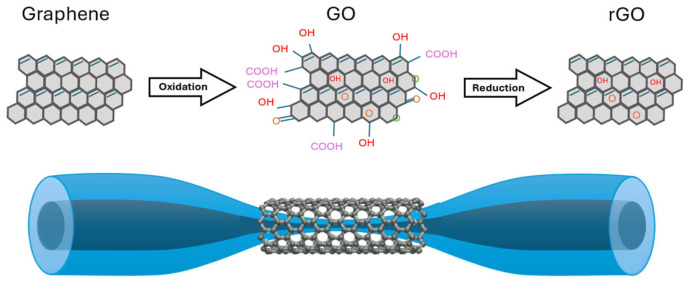
Scheme of the structural representations of graphene, GO, and rGO.

Graphene has recently attracted significant attention due to its unique properties, such as chemical stability, flexibility, high mechanical strength, and excellent thermal conductivity, which is among the best of all materials. Graphene exhibits a low absorption coefficient, which makes it well suited for integration with optical fibers with minimal losses, along with unique optical properties. The nanoscale structure of graphene offers an immense surface-to-volume ratio, enabling it to detect the smallest measurable gas levels (even a single molecule), making it an attractive candidate for chemical sensor applications [131]. Alternatively, graphene oxide (GO) also demonstrates promising detection capabilities. GO’s carbon–oxygen structure enhances hydrophilicity, which in turn increases sensitivity. GO layers have been utilized in humidity sensors, heavy-metal detection, and nitrogen dioxide (NO_2_) sensors. Consequently, graphene-based materials hold significant potential as functional materials when combined with optical fibers [131,132]. Reduced graphene oxide (rGO) is a graphene-like nanosheet obtained by chemically, electrochemically, or thermally reducing GO. Due to the removal of oxygen groups, rGO exhibits higher electrical and thermal conductivity than GO, with its properties varying depending on the reduction method [133]. Figure 12 presents the structural representations of graphene, GO, and rGO, as well as the scheme of graphene-based coatings for the TOF probe.

A magnesium sensor was developed using a 6 μm TOF coated with graphene. The efficiency of the TOF sensor with and without a graphene layer was compared. Experimental results showed that the output power decreased as the magnesium concentration increased. The bare TOF sensor had a sensitivity of 12.1 dB/% with slope linearity greater than 98.31% and a resolution of 0.0102%. However, when the TOF was coated with a graphene layer, sensitivity increased to 19.63 dBm/% with better slope linearity (99.25%) and resolution (0.0038%). Furthermore, the resonance peak shifted to a longer wavelength as the magnesium solution concentration increased at room temperature [39].

**Figure 13:**
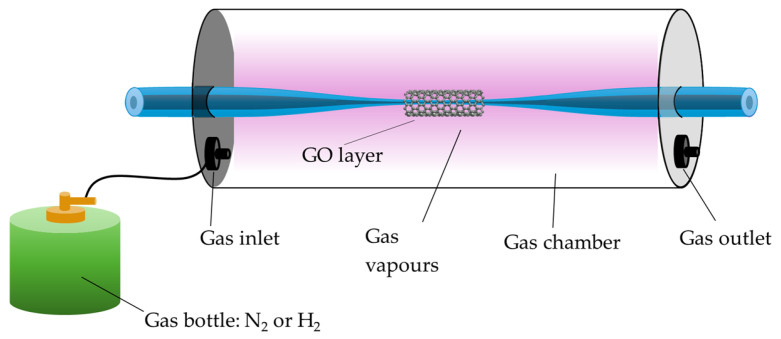
The scheme of the gas sensor based on a TOF covered with a GO layer.

GO, due to the presence of various functional groups such as -C-O-C-, -OH, -COOH, and -C=O, has excellent adsorption properties, which allow for the easy attachment of specific gases, such as CO, NO, NH_3_, or SO_2_ [133]. An example of such a sensor is presented in the work of [134], where a TOF covered with GO was used for detecting N_2_, H_2_, and LPG (propane–butane mixture). Based on the conducted studies, it was shown that for N_2_ and H_2_, the largest changes in power level resulting from gas adsorption were observed in the range of 750–850 nm, while for LPG, they were observed throughout the entire studied range of 500–1800 nm. The experiment is schematically presented in Figure 13 below. GO coatings can also be used to detect volatile liquids such as TMP, THX, and NH_4_OH [135].

There are also modifications of such sensors where the GO layer is doped with platinum nanoparticles (Pt NPs) to increase the sensor’s sensitivity. In one of the studies, the TOF was made from Ge_15_Sb_20_Se_65_ glass with a waist diameter of 50 μm and a waist length of 9.6 mm. The sensitivity of the TOF sensor modified with PtNPs/GO for monitoring ethanol was significantly increased, being 2.43 times higher than that of the GO-coated sensor and 7 times higher than that of the uncoated sensor [136]. 

Combinations of TOFs modified by PDMS combustion products and GO are also ideal for MUC1 (mucin 1) detection. In [137], a highly sensitive label-free biosensor fabricated using the flame-heated TOF drawing technique was presented, exhibiting a detection limit of 0.11 pM within the concentration range of 0–400 μg/mL MUC1. Hernaez et al. presented a TOF coated with GO and polyethylenimine (PEI), which was fabricated through the layer-by-layer technique and operated on the LMR principle. Their research involved two fibers covered with 8 and 20 bilayers of PEI-GO, which were analyzed both statically and dynamically as refractometric sensors [138].

The literature also describes the combination of GO with polymers to create humidity-sensitive coatings. One example is a humidity sensor developed by Syuhada et al. based on a non-adiabatic TOF coated with a GO and poly(vinyl alcohol) (PVA) nanocomposite film. The fabricated sensor demonstrated a sensitivity of 0.00624 ± 0.00033 a.u. (%)^−1^ when exposed to relative humidity (RH) levels ranging from 20% to 99.9% RH. The hybrid integration of GO and PVA improved the sensor’s sensitivity by approximately 15.86% compared to uncoated fibers [139].

The GO is also connected with Fe_3_O_4_ NPs for magnetic field detection. The sensor was characterized by a high sensitivity of 99–324 pW/mT. The parameters of the used TOF were 6.8–11 mm in length and 3.3–12.8 μm in diameter [140].

An interesting structure was proposed by Fan et al. [137,138], introducing a novel approach where two [141], three [142], or four [142] TOFs were twisted together. A graphene coating was applied to this structure, enabling the development of temperature and strain sensors. The parameters of sensors were, appropriately, sensitivities of −66.2 pm/°C and −173.4 pm/με for three-twisted TOFs, sensitivities of −61.7 pm/°C and −103.2 pm/με for four-twisted TOFs, and sensitivities of −127.2 pm/°C and −173.9 pm/με for two-twisted TOFs. 

Based on the analyzed literature, GO is the most commonly used material in the aforementioned study. This is due to its high surface reactivity, excellent electrical conductivity, large surface area, and outstanding mechanical strength. Moreover, recent studies highlight a growing trend toward hybrid solutions that combine GO layers with NPs of metal and polymer layers, which effectively enhance selectivity and enable the development of specialized sensors.

### 3.2. Loop, Knot, and Microcoil Sensors as Microring Resonators

The TOF technology, which has been mastered and perfected, now allows the production of fibers with diameters as small as a few micrometers or even nanometers. According to the previously described theory, light propagating within the fiber is partially exposed to the external environment as an EW. The small diameter of micro-/nanofibers ensures a large EF [41], making these structures highly suitable as sensor bases. Moreover, the length of the TOF is sufficiently large to allow for the formation of different geometric configurations. Usually, this group of sensors is called microring resonators. The most well-known structures of ring resonators are microloop resonators (MLRs), microknot resonators (MKRs), and microcoil resonators (MCRs). In the literature, these types of sensors are described by parameters such as quality factor (*Q-factor*), finesse *f*, and the Free Spectral Range (FSR) [41].

**Figure 14:**
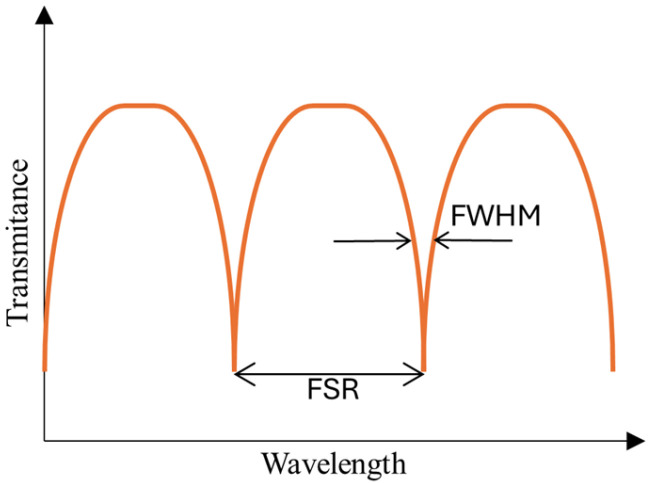
Schematic diagram of the spectral characteristics of ring resonators with characteristic values for the marked structure.

The *Q-factor* is defined as follows (see Figure 14) [41]:

(9) $$Q=\frac{\lambda_{res}}{FWHM}.$$

where $\lambda_{res}$ is the resonant wavelength and FWHM is the spectral full width at half maximum of the resonance peak, expressed in wavelength units [143].

(10) $$FSR\approx \frac{c}{{2n}_{eff}L}.$$

In this equation, *c* is the speed of light, *n_eff_* is the effective RI of the propagated mode, and *L* is the length of the loop [143]. The last parameter *f* is described as follows [143]:

(11) $$f=\frac{FSR}{FWHM}.$$

**Figure 15:**
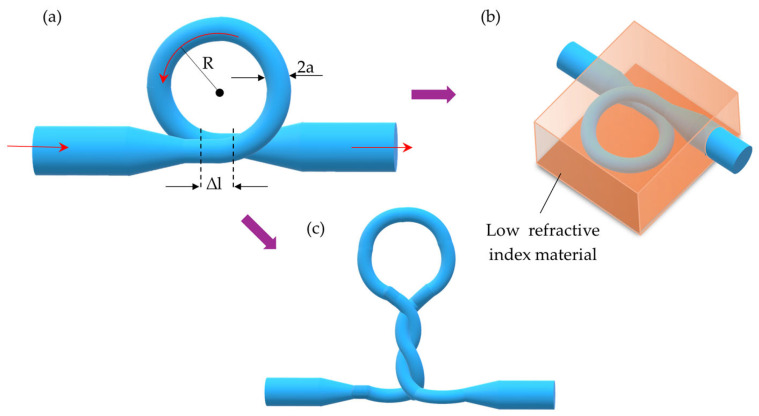
Structure of an (**a**) MLR and its parameters, (**b**) an embedded MLR, and (**c**) a twisted MLR.

The first type of resonator structure is an MLR. This structure is stabilized by van der Waals interactions and electrostatic forces at the TOF interface (Figure 15a) [41,144]. The literature describes geometric parameters of MLRs that affect the parameters of the sensors. The key parameters considered include a diameter of TOF *2a*, an overlap region/coupling region Δ*l*, and a loop radius *R* [34,144]. These parameters directly affect sensor properties such as sensitivity, *Q-factor*, the detection limit, and the FSR of the sensor. Shi et al. [144] conducted simulations to analyze the impact of these parameters on sensor performance, with the results presented in Table 3.

Due to the low mechanical stability of these structures, they should be reinforced using additional materials (e.g., polymer or glass) with a low RI (see Figure 15b). This type of structure is known as embedded micro-/nanofibers [145]. Cai et al. [146] proposed an MLR structure enclosed in PDMS. Another method for improving stability involves twisting the MLR, as shown in Figure 15c. This modification increases Δ*l* (overlap region), which directly improves the *Q-factor* [147]. Sun et al. [147] implemented this modification, achieving a high sensor sensitivity of 24,373 nm per RI unit and temperature stability around 0.005 nm/°C.

Jali et al. developed an MLR sensor with a loop diameter of 300 μm and a taper waist diameter of 7 μm. The sensor’s sensitivity to RI changes was evaluated based on the phosphoraldehyde concentration (0–5%). Compared to a sensor using only a straight microfiber, the MLR sensor demonstrated 2.5 times higher sensitivity and 3.28 times better resolution [148]. The MLR structure can also be used as a sensor for measuring gas pressure. In 2022, simulations were conducted for an MLR-based sensor designed to measure the pressure of O_2_, CO_2_, and He, the sensitivity of which varied depending on the gas used, which ensures its selectivity [34].

**Figure 16:**
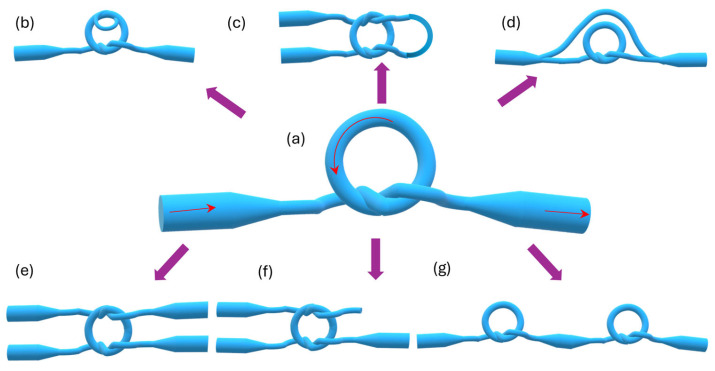
Modifications of the MKR: (**a**) structure of the MKR, (**b**) parallel double knot, (**c**) an MKR as a U-shaped interferometer, (**d**) MKR coupling with another bent microfiber, (**e**) square resonator, (**f**) reef knot, and (**g**) dual microfiber knot resonators (DMKRs).

To enhance the durability of MLRs, these structures can be modified by tying the loop into a knot [149]. Compared to a simple loop, the geometry of the resonator is maintained by electrostatic forces and friction between the knot segments (Figure 16a), where the overlap region can be regarded as a coupler. This structure was used in a variety of sensors, including temperature [150], humidity [151], strain [152], and RI [150] sensors. Yue-Yu et al. proposed a microfiber knot resonator embedded in a high-birefringence fiber loop mirror (HB-FLM), allowing for simultaneous measurements of temperature and the RI. The conducted research allowed for the determination of the sensitivity of HB-FLM to a temperature of −464.1 pm/°C for the fiber loop mirror and 5.6 pm/°C for MKRs, while the RI of dilute solutions was determined at 116 nm/RIU [153].

Sensors utilizing MKRs perform exceptionally well as acoustic sensors. One example is the underwater acoustic sensor with PDMS coating, which was presented in 2024 by Zhai et al. The fabricated sensor contained a microfiber with a diameter of 3.5 μm, tied into a knot with a diameter of 1.1 mm, achieving an average sensitivity of −135.8 dB within the frequency range of 10 Hz to 2 kHz and an average signal-to-noise ratio of 53.2 dB. [154]. The same authors proposed ultrasonic underwater detection using a microfiber knot resonator based on the whispering gallery mode (WGM) principle. In this case, they achieved a sensitivity of −173.2 dB within the frequency range of 180 kHz to 1 MHz and a minimum detectable sound pressure of 0.046 mPa/√Hz [155]. Another group of authors from Yanshan University also obtained an ultrasound sensor with an SNR maximal value of 37.89 dB from 25 kHz to 305 kHz [156].

MKRs can be used to detect longitudinal load changes. For example, Cai and Li, in their paper, presented a strain sensor based on an MKR packaged with PDMS, which contained a micro-/nanofiber with a diameter of 6.92 μm and a microring diameter of 6 mm. The fabricated sensor exhibited a strain sensitivity of 94.5 pm/N [152]. On the other hand, another study, by Tian et al., utilized this optical fiber element for ambient relative humidity measurement. One of the advantages of the fabricated micro-hygrometer sensor is that it does not require any coating with hygroscopic materials. Additionally, the obtained RH sensitivity reaches 5.95 pm/% RH within the linear range of 30% RH to 94% RH [151].

One of the most intriguing MKR modifications is the microfiber double-knot resonator with a parallel structure (see Figure 16b). A theoretical study on gas RIs was conducted by a research team from Yangtze University. They performed simulations to explore the feasibility of detecting the volume content of hybrid CO and CO_2_ gases. Their findings demonstrated that microfiber diameter significantly impacts gas sensitivity. For a 1.0 μm diameter, the sensor achieved a maximum sensitivity of 98.4 nm/RIU [157].

Figure 16c presents a U-shaped MZI created based on an MKR. This structure was proposed by Yi in 2022 as a humidity sensor [158]. The sensitivity was 2.442 nm/%RH with a measurement range from 60% RH to 95% RH. The Vernier effect mechanism was used. The functional material agarose was applied to the sensing part of the microfiber.

Another modification structure of the MKR was shown by Chen (Figure 16d). The MKR was connected with a straight microfiber and created an MZI. The proposed result was characterized by a higher *Q-factor* and higher extinction ratio than the standard MZI [159].

An interesting structure is a square resonator (see Figure 16e), which consists of two coupling areas. The two MKRs are connected together [160]. The main conclusion is that if the structure is more complex, then the losses increase. The article compared five different configurations of MKRs.

A reef knot (Figure 16f) was proposed by Vienne et al. [161]. In the article, two MKRs were used to create this structure. The MKRs differed in the material they were made of, namely, silica and chalcogenide microfiber.

Another advanced modification is the dual microfiber knot resonator (DMKR), which consists of two MKRs cascaded in series (Figure 16g). This sensor works on the principle of the Vernier effect and allows the simultaneous sensing of RIs and temperature. In the study [150] conducted by Yang et al., the fabricated sensor exhibited an RI sensitivity of −12523 nm/RIU (in the range from 1.3375 to 1.3359), which is 40 times higher than that of a single MKR, while the temperature sensitivity reached 0.91998 nm/°C (in the range from 0 °C to 25.3 °C).

**Figure 17:**
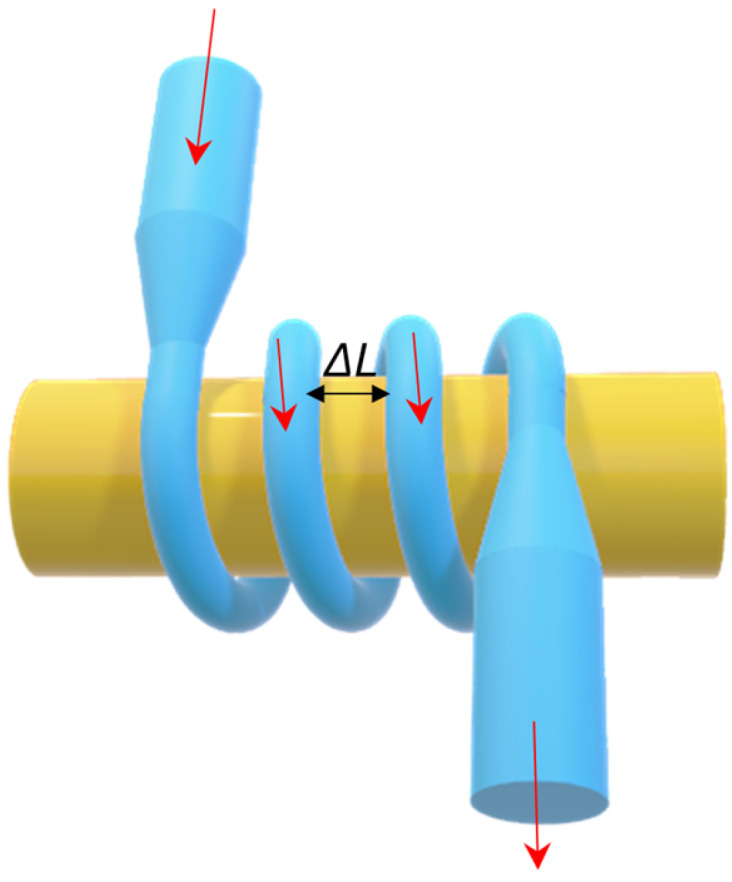
Structure of MCR.

Another structure based on a nanometer-sized TOF is the MCR (see Figure 17). This is created by wrapping TOFs around a rod. The rod is characterized by a low RI. If successive coils are placed close enough together, light can couple between them. As a result, the *Q-factor* is high in the range of 10^10^ [162]. Theoretical considerations were first presented by Sumetsky et al. [162]. Another mathematical model was presented by Xu et al. [163]. An additional advantage of such structures is significantly increased stability and coupling areas. Moreover, such a configuration allows for an easier manipulation of resonance parameters and sensitivity only by changing the radius or material of the rod on which the constriction is wound, as well as by changing the number of turns and the distance between them. The influence of the modification of individual properties on the MCR resonance parameters is presented in Table 4 [164].

Chen et al. proposed MCRs as temperature sensors [165]. The micro-TOF was wound up around a Teflon rod. The sensor was characterized by a responsivity of 95 pm/°C between 26 and 76 °C. Isameel, in 2012, modified MCR structures and applied a microfiber coupler wrapped around a polymer rod [166]. The MCR was also proposed by Wang et al. [35]. The MCR structure was functionalized with MXene, and a polycarbonate rod was used. As a result, this structure can be applied as a tunable filter or an optical modulator. A magnetic field sensor was proposed by Talataisong [167]. The MCR sensor was embedded in a polymer. The device was characterized by a magnetic field sensitivity of 37.09 dB/T and a detection limit (DL) of around 27 μT.

When analyzing the overview of resonator structures, it can be seen that the MKR structure is most often chosen by researchers. A lot of modifications to this structure have been made. The reason for this may be that this structure is characterized by high stability in relation to MLRs and easier technology in relation to MCRs. The new structures of resonators based on TOFs are Microbottle Resonators (MBRs) [168], which are characterized by high *Q-factors*. These structures have huge potential to be applied in future sensing applications.

### 3.3. Fiber Bragg Gratings

**Figure 18:**
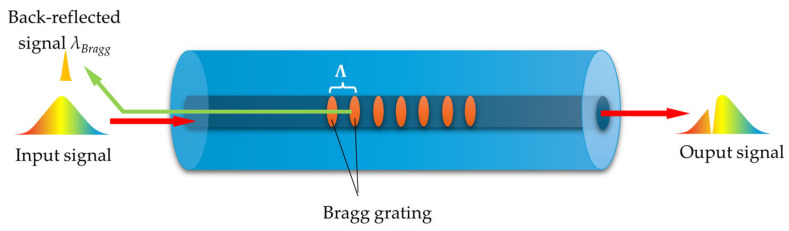
Scheme of the operating principle of the FBG.

In recent decades, fiber Bragg gratings (FBGs) have garnered significant research interest in the field of optical sensing. Numerous FBG-based sensors have been developed to measure various parameters, including temperature [169,170], strain [169,170,171], seismic detection [172], torsion [170], pressure [173], tilt angle [174], acceleration [175], and force [176]. They consist of a periodic structure characterized by variations in the RI of the fiber core. When light encounters an interface between materials with different RIs, it undergoes reflection and refraction. The fundamental working principle of an FBG relies on the Fresnel reflection that occurs when light encounters an interface between two materials with different RIs. At this boundary, a portion of the light is reflected back, while the rest is transmitted and refracted according to Snell’s law [177]. The amount of reflected light depends on the RI contrast between the two media and the angle of incidence. The scheme of the FBG sensor’s working principle is presented in Figure 18, where the alternating RI pattern due to the presence of the Bragg grating is marked in orange, and its periodicity is denoted as Λ. The periodic variation in the RI within the fiber core creates multiple interfaces where partial reflections occur. When these reflections constructively interfere, they reinforce a specific wavelength known as the Bragg wavelength, *λ_Bragg_*, while other wavelengths pass through with minimal reflection [177]. In a uniform FBG, the central wavelength of the reflected spectrum is approximately equal to the *λ_Bragg_*, which is defined by the following:

(12) $$\lambda_{Bragg}=n_{eff}\Lambda$$

where *n_eff_* represents the effective RI of the guided mode and Λ denotes the grating’s spatial period.

There are several methods for producing Bragg gratings in optical fibers, with one of the most commonly used being direct writing through a periodic (or non-periodic) phase mask using UV light [178]. During the exposure process, the fiber is placed a few micrometers away from the mask, and the radiation passing through the mask undergoes diffraction of various orders (0, ±1, ±2, etc.) [179]. The resulting RI perturbations introduce a significant path for fiber optic sensing due to the tunable structure of the grating. One of the main advantages of this method is that the periodicity of the grating is independent of the angle of incidence and the wavelength of the UV beam if it is less than 10 degrees [180]. Additionally, the grating fabrication process is much faster compared to other methods.

Another approach for introducing periodic RI modulation is the point-by-point inscription method. This method involves exposing a single point of the fiber to a UV beam at a time using a low-numerical-aperture lens and short laser pulses [169,181]. After each exposure, the fiber is moved to the next point. However, this method requires a translation stage with extremely high precision, and the grating fabrication process is relatively time-consuming.

The last method is the holographic technique, which uses either a solid-path or free-space interferometer [182]. This approach involves creating a grating by focusing an interference pattern onto the fiber core. The periodicity of the grating can be controlled by adjusting the angle between the interfering beams or by changing the wavelength of the light used [183].

A very promising method for creating Bragg grids is the use of femtosecond lasers, which can be applied in phase mask, direct writing, and holographic methods. The main advantage of using these lasers is to make the process more efficient and precise, allowing for a significant reduction in the thermal effects associated with the writing process [178,184,185].

#### 3.3.1. FBGs and LPGs

**Figure 19:**
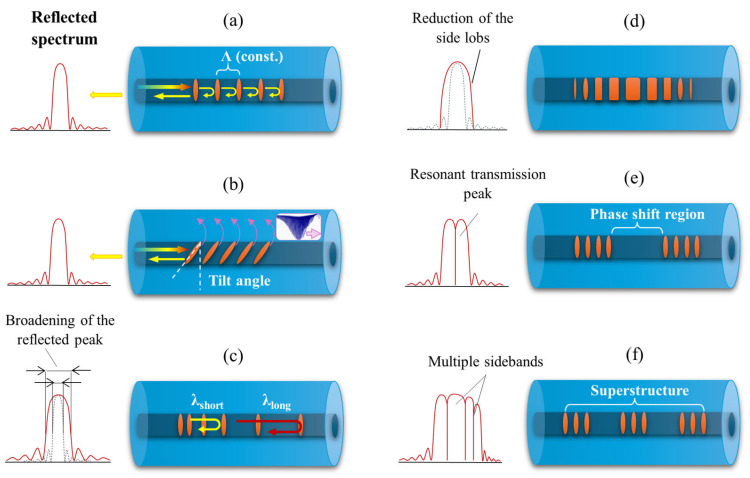
Typical types of FBGs: (**a**) standard FBG with uniform spatial period Λ below ~0.5 μm; (**b**) tilted; (**c**) chirped; (**d**) Gaussian-apodized; (**e**) phase-shifted, and (**f**) superstructure.

As mentioned earlier, one of the key parameters characterizing gratings is their periodicity, which determines their working principles. For this reason, they can be divided into two main types: short-period gratings (SPGs) with a grating period up to 0.5 μm and long-period gratings (LPGs) whose periodicity can reach up to 1 mm. As described earlier, SPGs operate based on the Fresnel reflection according to the Bragg condition, while LPGs do not reflect light but couple the core-guided light to the cladding modes. After coupling, the modes propagating in the cladding are rapidly attenuated due to scattering and absorption by the surrounding medium. Since propagation does not occur based on total internal reflection, they cannot be effectively guided [186]. This is why transmission spectra of LPGs show attenuation peaks corresponding to wavelengths that have been coupled to the cladding modes and simultaneously phase-matched with the LPG’s period. In Figure 19 below, the most popular types of FBGs are schematically presented, while Table 5 contains a short comparison of FBGs and LPGs in relation to features and working principles.

Among the short-period gratings, several types can be distinguished:

- Gratings with a constant period and perpendicular perturbation of the RI relative to the fiber axis (Figure 19a): However, such gratings may generate undesirable reflections at different wavelengths, which interfere with data transmission.
- Tilted where the RI perturbation is rotated by a certain angle θ, as shown in Figure 19b: Depending on the tilt angle of the grating, coupling of the core mode to several selected cladding modes may occur, which can lead to modifications in the position and shape of the reflected peak. This process can simultaneously significantly improve the resolution of the sensor [187].
- Chirped, where the period is not constant along the entire length of the grating but changes with distance: These gratings operate in the dispersion compensation polarization mode, and different wavelengths are reflected at different locations on the grating [188] (Figure 19c).
- Gaussian-apodized, meaning a grating with a Gaussian profile: This reduces the effect of side lobes in the reflection, which could interfere with the signal in optical fiber systems [189] (Figure 19d).
- Phase-shifted, where the grating introduces a phase shift at one or more points in the grating: This results in the formation of a resonant transmission peak—a very narrow wavelength that can pass through the grating instead of being reflected [171] (Figure 19e).
- Superstructure grating, which refers to a periodic modulation along the fiber’s length: This is introduced through a sampling method, which results in the creation of sidebands in the reflection spectrum [169] (Figure 19f).

Recently, gratings that combine the features (structures) of several gratings have simultaneously become popular. An example is the combination of a superstructure and LPG, which creates a highly sensitive RI sensor. This proposed structure consists of periodically spliced no-core fibers and SMFs arranged based on a specific modulation function. This configuration efficiently excites higher-order cladding modes, enhancing RI sensitivity. Experimental findings demonstrate that the sensor exhibits a high RI sensitivity of 557.74 nm/RIU within the measurement range of 1.33 to 1.42 [186]. Another example is the use of an FBG containing a chirped and tilted grating to suppress the 1030 nm amplified spontaneous emission of a 980 nm ytterbium-doped fiber laser. By using a single CTFBG in the cavity of a 20/80 μm 980 nm YDFL, an output power of 6.4 W was achieved, along with a slope efficiency of 27% [190]. In the work by Cai et al., an innovative sensor is presented—a small-period long-period fiber grating (SP-LPG) for the simultaneous detection of D-glucose and temperature, based on the immobilization of concanavalin A on polydopamine nanospheres. The sensor demonstrated high selectivity and sensitivity for D-glucose detection (10 nM–10 mM) with a low detection limit of 1.95 nM (S/N = 3). Additionally, the small grating period of the SP-LPG enables the Bragg reflection for temperature sensing, achieving a thermal sensitivity of 10 pm/°C (30–100 °C) while eliminating temperature cross-sensitivity in RI measurements [191]. A recent study presents a superstructured CTFBG cascaded with a conventional FBG for the simultaneous measurement of temperature and the RI [192]. The sensor exhibits temperature sensitivities of 13.9 pm/°C for the CTFBG and 13.7 pm/°C for the FBG over a range of 25–300 °C. Additionally, the CTFBG shows an RI sensitivity of 1.8487 nm/RIU within the RI range of 1.3399–1.3648.

#### 3.3.2. Tapered Fiber Bragg Gratings 

Typically, Bragg gratings are made on standard SMFs or MMFs. However, using a Bragg grating on a tapered fiber (TFBG) can significantly enhance its sensitivity to external factors, especially to changes in the surrounding RI. This method integrates the benefits of conventional Bragg gratings with the features of TOFs, leading to distinct spectral and dispersion characteristics primarily shaped by the tapered profile. The structure of TFBGs differs significantly from other types of FBGs, and the process of writing them is complex and challenging. Unlike standard FBGs, TFBGs exhibit a different response to strain and temperature. This enables the simultaneous measurement of both parameters and allows TFBGs to function as strain sensors independent of temperature. As a result, they are widely used in sensing applications and dispersion compensation. However, TFBGs have limitations, including a narrow spectral width (typically not exceeding 3–4 nm) and a non-monotonic strain response [193,194].

To minimize the effects of low spectral width, a method for fabricating a structure based on linearly chirped TFBGs was proposed in 2016. This approach combines the effects of both the taper profile and a linearly chirped grating. As a result, it enables both a significant reduction in (in TCFBGs with counter-directional chirps) and broadening (in TCFBGs with co-directional chirps) of the reflected spectrum, which was previously unattainable in FBGs written in TOFs [195]. TFBGs are most commonly used as temperature sensors. An example is the asymmetric TFBG sensor fabricated using a femtosecond laser, which was proposed by Zhang et al. In their study, a TOF with a 25 μm diameter and 1000 μm length was used, and the FBG was directly written point by point using a femtosecond laser, with a grating length of 1000 μm. Within the temperature range of 30–350 °C, the obtained sensor exhibited a sensitivity of approximately 12.3 pm/°C [196]. In 2021, Afroozeh also proposed a temperature sensor with a sensitivity of 717 1/°C in the temperature range of 0–140 °C and a Bragg wavelength shift of 9.6 nm/°C. This value significantly exceeds the performance of sensors based on other fiber optic elements, such as D-shaped PCFs (10.61 1/°C in the range of 0–60 °C), Sagnac interferometers (1.65 1/°C in the range of 25–33 °C), or MZ interferometers (1.83 1/°C in the range of 23.2–58.2 °C) [197].

Recently, a team from the Nanjing University of Aeronautics and Astronautics proposed transforming a commercial FBG sensor based on SMFs into a microfiber using the hydrothermal method with NaOH and HCl. This approach allows for a significantly smoother surface compared to the use of HF. The method is suitable for batch production, as it achieved a surface roughness of approximately 87 nm and enabled the fabrication of tailored fiber diameters with an error of only ±1.2918 μm [198].

**Figure 20:**
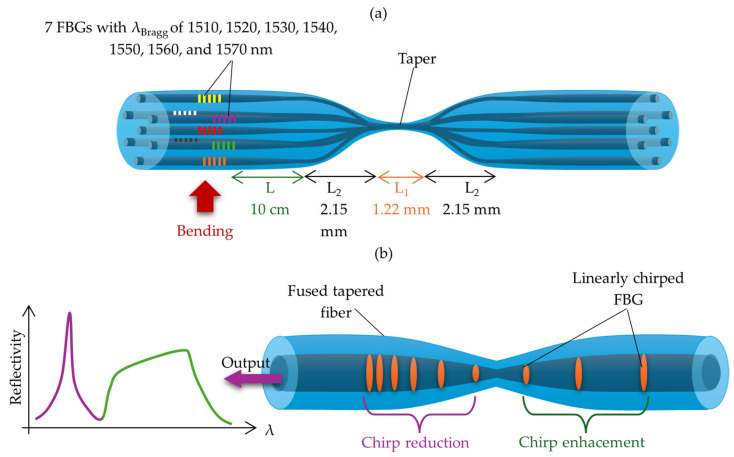
Scheme of the TFBG sensors of (**a**) vector bending as well as (**b**) strain and temperature.

Another interesting solution is the development of a vector bending sensor that enables the simultaneous measurement of the bending response in the six outer cores of a seven-core fiber (SCF), where seven separate Bragg gratings are inscribed across the fiber. The FBGs were written point by point in all seven cores, with Bragg wavelengths of 1510, 1520, 1530, 1540, 1550, 1560, and 1570 nm. Subsequently, a TOF was introduced approximately L = 10 cm away from the inscribed gratings (see Figure 20a). This approach allowed for the bending response of the sensor to be obtained, achieving a maximum sensitivity of 127 pm/m^−1^ [199]. Another example of a TFBG used for strain measurements is the Fabry–Perot cavity based on chirped TFBG presented by Markowski et al. The structure included two linearly chirped fiber Bragg gratings with a chirp rate equal to 0.35 nm/mm that were positioned next to each other at a tapered transition region in such a way as to achieve a matching condition for both Bragg wavelengths [200] (Figure 20b). The applied sensing mechanism enabled a strain sensitivity of 5 μm/με and a temperature sensitivity of 8.96 pm/°C.

**Figure 21:**
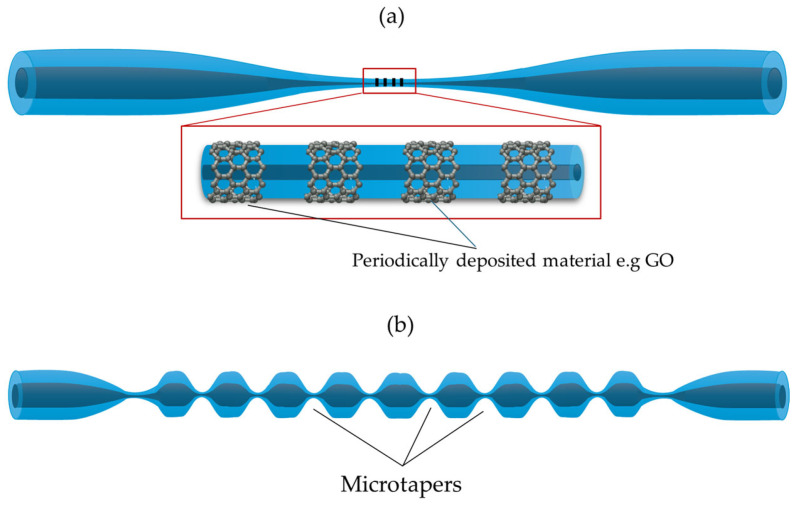
FGBs made by (**a**) periodically depositing functional materials on the TOF area and (**b**) micro-tapering the LPFG.

Another approach involves creating gratings by periodically depositing functional materials onto the TOF. Commonly used materials include polymers [201,202] and GO [203,204] (see Figure 21a). One of the major advantages of this type of sensor is its extremely high sensitivity—up to 10,419 nm/RIU in the RI range of 1.4102–1.4164 [201] and 11,605.79 nm/RIU in the RI range of 1.4558–1.4577 [203].

In addition to this method, micro-tapered LPFGs have also been explored. The main distinction lies in the fabrication process: the grating is produced by periodically tapering a standard optical fiber, rather than forming it directly on a TOF [205,206] (Figure 21b). This technique is considered simpler and provides greater mechanical stability while maintaining favorable sensor performance.

**Figure 22:**
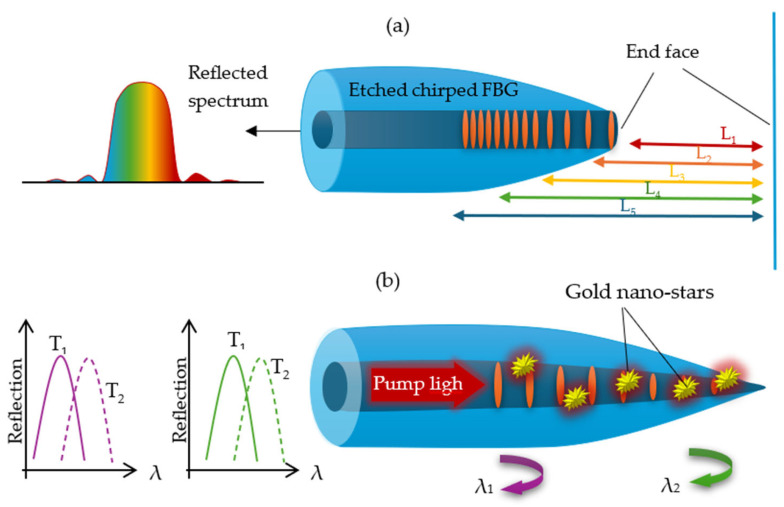
Examples of FBG tip sensors: (**a**) chirped fiber tip, which serves as a Fabry–Perot interferometer; (**b**) tip covered with gold nanostars, which serves as converters of light into heat.

TFBGs can also be fabricated in the form of a tip. Such a solution can be found in [36], where a chirped fiber tip serves as a Fabry–Perot interferometer. The discussed structure consists of an etched chirped fiber Bragg grating (FBG) and a broadband end-face mirror at the fiber tip (Figure 22a). As a result, different resonance wavelengths correspond to various grating positions, leading to a gradient in the cavity length, and this FP interferometer exhibits a chirped interference spectrum. Experimental results show that the dips in the reflection spectrum of the FP interferometer have similar temperature sensitivities (~11 pm/°C) within the temperature range of 25 °C to 65 °C [36].

In another study [207], gold nanostars were used to enhance the on-fiber photothermal effect and plasmonic tunability (see Figure 22b). The Bragg grating was written into a TOF to obtain a diameter-dependent spectrum that utilized chirped signals. The photothermal effect was realized by attaching gold nanostars to the fiber surface, which served as converters of light into heat. As a result, a sensor for gradient temperature sensing was developed, with a photothermal efficiency of 0.21 °C/mW, while maintaining a localized temperature increase of up to 60 °C in aqueous environments.

It can be observed that gratings are increasingly combined with various functional materials to effectively utilize phenomena such as SPR—involving metals—and the enhanced interaction between the EW and the sensing medium, for example, through the use of graphene. Additionally, recent research has focused on the development of FBG and LPG superstructures. These advanced configurations offer a wide range of design combinations that can significantly influence sensor performance. A key conclusion is that future research should aim to integrate the benefits of multiple grating types—such as chirped and tilted gratings—simultaneously, in order to overcome the limitations of standard structures and to optimize sensor sensitivity [208].

## 4. Summary and Future Trends

This review presents numerous possible combinations of TOF-based structures, which work excellently as simple and highly sensitive optical sensors for various physical, chemical, and biological factors. Table 6 compiles and presents examples of the latest TOF-based sensors along with their working principle and measured sensitivity to the detected factors.

By analyzing Table 6, it can be observed that a variety of possibilities and potential changes can be implemented in TOF structures, which can be briefly summarized below:

- The TOF structure can be shaped by changing its dimensions and geometry;
- A variety of configuration sensors, like a straight TOF, TOFs as a part of interferometers, and TOFs with FBGs and LPGs, can be observed;
- More and more often, materials are combined in a hybrid form (two or three materials) to improve the parameters of the designed sensor;
- Depending on the coating material, different phenomena enable the detection of the measured parameter: SPR, LSPR, LMS, etc.

**Figure 23:**
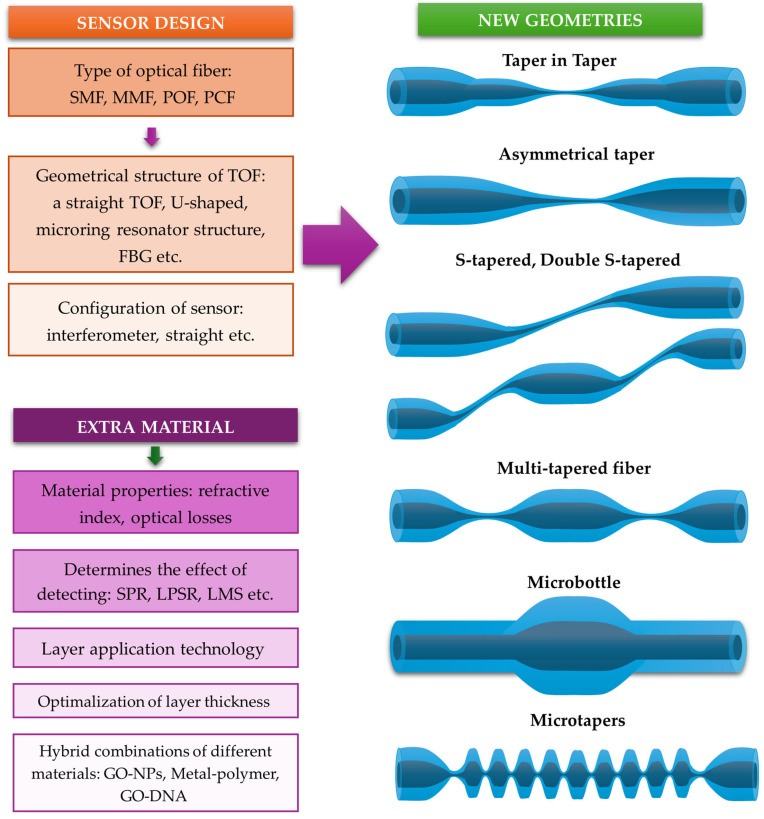
Process of optimization of TOF sensors and new trend in geometric structures.

Due to the continuous development of modern fabrication methods, TOF structures remain highly popular among researchers. An increasing number of new geometries and advanced structures (Figure 23), such as TIT structures [106,107,128], S-shaped TOFs [108,211,212], multi-tapered structures [213,214], micro-tapered LPFGs [215], gratings based on extra material [203], asymmetrical tapers [216] and MBRs [168], can be observed (Figure 23). Each of these modifications brings a change in the working principle or parameter that the structure uses for characterization. The first one, denoted as the TIT structure, works in the mode–mode interference principle [128]. TIT structures are characterized by several geometric parameters, such as the first and second diameters of the taper waist, the second taper length, and the total length of the sensor. Each of these parameters directly affects the sensitivity of the resulting sensor. In such structures, as light propagates through the fiber and reaches the first and second tapers, many high-order modes are excited, while part of the light leaks out, forming an EF around the TIT structure. Then, as all modes recouple back into the fiber, a specific pattern in the spectrum can be observed.

Certain structures, such as S-tapered [212] and double S-tapered fibers [211], are typically presented in the MZI configuration. In both cases, when designing sensors, the gradient angle must be considered, whereas in the case of double S-tapered fibers, an additional parameter is the distance between consecutive tapers. The main difference in light propagation, compared to TOF structures, is that when light passes through a tapered S-shaped fiber, some of it leaks from the core into the cladding and then recouples back into the core, resulting in intermodal interference.

Asymmetric TOFs [216] are characterized by two tapered transition regions: the first, which is long, forms an adiabatic TOF, while the second, shorter region creates a non-adiabatic TOF. The presented structure functions as a Michelson interferometer operating in a reflective mode, with the interference signal arising between core and cladding modes [216].

In the case of multi-tapered sensors [213,214], the main parameters that characterize the structure are the taper waist diameter, the number of tapers, and the spacing between them. The operating principle is based on mode–mode interference, where the presence of multiple tapers causes changes in the optical path lengths of the higher-order cladding modes and the fundamental core mode.

Recent studies show that FBG- and LPG-based structures utilizing TOFs continue to be developed and refined, for example, by incorporating additional functional materials or introducing periodic nodes [203,215], as well as by integrating them with micro-tapered LPFGs. Compared to multi-tapered configurations, micro-tapered LPFGs contain a higher number of closely spaced TOFs. These structures exhibit very high sensitivity to changes in the external RI, making them suitable for sensing various physical and chemical parameters. Additionally, superstructures are being developed by combining two or more gratings with different physical characteristics—such as CTFBGs [190], SP-LPGs [191], or CTFBGs cascaded with FBGs [192]—as well as by integrating FBGs with interferometric structures [215].

Among the various types of resonant structures, MKRs are among the most rapidly developing. Compared to other resonator types such as MLRs and MCRs, MKRs offer several advantages: they are easier to fabricate, exhibit greater structural stability, and provide more flexibility for geometric modifications and integration with functional materials. Another recently introduced resonator structure is the MBR, which is fabricated on a TOF by locally varying the fiber radius using the soften-and-compress technique (Figure 23). The operating principle of MBRs is based on the total internal reflection of the light coupled into the structure. Additionally, MBRs are capable of supporting whispering gallery modes (WGMs) [168].

Sensors based on TOFs show great potential for sensing applications due to their high sensitivity to changes in environmental conditions. However, it is important to recognize the limitations of these sensors and to adapt their structures for future applications. One of the main limitations of TOF-based sensors is their low mechanical stability. Due to their small dimensions, TOFs are fragile, and integrating them with additional materials can be technically challenging. Furthermore, when selecting functional materials, their optical properties must be carefully considered. These include an appropriate RI, optimal coating thickness to control optical losses, and compatibility with the desired operating wavelength range.

Currently, among various types of coatings, gold, metal oxides, and polymers are most commonly used. However, continuous layers of these materials are typically rigid, which makes the TOF sensors that are covered with them fragile. For this reason, in recent years, nanomaterials such as NPs, quantum dots, and nanotubes have been proposed as coating components to improve sensor performance. To further enhance their sensing capabilities and to introduce selectivity toward specific analytes, hybrid coatings like polymers with metals and graphene with NPs are also frequently employed. Nevertheless, some types of TOF sensor structures face limitations for commercial applications related to their fragility and long-term stability.

The diagram above illustrates the complex process of sensor design, which involves selecting various parameters, such as the type of optical fiber, design, and coating materials, all of which directly affect the sensor’s performance and characteristics (Figure 23).

## 5. Conclusions

Significant potential for future applications is held by sensors based on tapered optical fibers with additional coatings. This review was initiated with an outline of the methods used for fabricating tapered fibers, followed by an explanation of evanescent field principles and mode evolution. A detailed analysis was then provided of fiber optic tapers coated with functional materials.

The surface plasmon resonance effect in metal-coated tapers was explored, and various sensor designs utilizing thin metal films, metal nanoparticles, and their combinations for chemical and biological detection were discussed. Subsequently, oxide coatings were introduced, and the localized mode resonance phenomenon—serving as a fundamental principle in sensors incorporating oxides, polymers, and graphene-based layers—was examined. Functional coatings such as polymers, organic layers, and various graphene-based structures were also reviewed.

In addition to material coatings, the geometric configurations of microfibers were analyzed, including loops, knots, microcoils, fiber Bragg gratings, and long-period gratings, and their diverse sensing applications were highlighted.

In conclusion, TOF-based sensors with advanced coatings are being recognized as a rapidly evolving field. Advances in taper fabrication techniques and layer deposition methods have enabled the development of highly sensitive and application-specific sensors. The continuous emergence of new research is being viewed as a sign of the vast potential of this technology, paving the way for future innovations in chemical, biological, and environmental sensing.

## Figures and Tables

**Table 1:** Dielectric constants and the approximate operating wavelength range of the sensor containing a given metal as a cover.

Metal	ε_r_ + iε_i_ *	ε_r_/ε_i_	Operating Wavelength Range	Refs.
Au	−11.36 + 1.23i	9.23	VIS	[72,73]
Ag	−18.22 + 0.48i	37.9	VIS	[74,75]
Cu	−12.46 + 0.65i	19.2	VIS-IR	[76,77]
Al	−43.42 + 15.40i	2.82	UV-NIR	[78,79]
Bi	−23.44 + 2.18i	10.7	UV-NIR	[80,81]
Pd	−15.27 + 15.17i	1.01	NIR-IR	[82,83]

Ranges: VIS—visible; IR—infrared; UV—ultraviolet; NIR—near-infrared; ε_r_—real part of dielectric permittivity; iε_i_—imaginary part of dielectric permittivity; ε_r_/ε_i_—ratio of dielectric constants; *—determined for the wavelength λ = 632.8 nm.

**Table 2:** Values of the dielectric permittivity of ITO, TiO_2,_ and Al_2_O_3_. The dielectric permittivity of SiO_2_ is given as a reference.

Material	ε_r_ + i*ε*_i_ *	Ref.
SiO_2_	2.08 + 0.000058i	[98]
ITO	3.24 + 0.01i	[99]
TiO_2_	5.92 + 0.00048i	[98]
Al_2_O_3_	3.15 + 0.0078i	[100]

* Determined for the wavelength λ = 632.8 nm; ε_r_ and iε_i_—real and imaginary part of permittivity.

**Table 3:** Changes in the properties of the sensor depending on the geometrical parameters of an MLR [144].

Properties of Sensor	Geometric Parameter on Which It Depends	Dimension
High Sensitivity	*2a*	small
	hardly dependent on *R*	small
Higher *Q-Factor*	*2a*	large
	*R*	large
	Δ*l*	appropriate
Lower Detection Limit Factor	*2a*	large
	*R*	large
	Δ*l*	appropriate
Large FSR—Related to Wide Dynamic Measurement Range	*2a*	small
	hardly dependent on *R*	small

*2a*—a diameter of TOF; Δ*l*—overlap region/coupling region; *R*—loop radius.

**Table 4:** Influence of changes in MCR parameters on resonance properties [164].

Modified Parameter	Modification Increasing (↑)/Decreasing (↓)	Effect on Resonant Parameter	
		Parameter	Effect
Diameter of the supporting rod	↑	FSR	↓
Gap between the coils	↓	FWHM	↓
		*Q-factor*	↑
		E_r_ *	↑
Number of coils	↑	FWHM	↓
		*Q-factor*	↑
Optical loss coefficient	↑	E_r_*	↑

* Extinction ratio (E_r_).

**Table 5:** Comparison of FBGs and LPGs in relation to features and working principles.

Feature	LPG	FBG
Working principle	Coupling of core modes to cladding modes	Fresnel reflection according to the Bragg condition
Grating period	100–1000 μm	~0.5 μm
Operating mode	Transmission (light passes through the grating)	Reflection (light reflects from the grating)
Bands in the spectrum	Losses in the transmission spectrum	Reflection in the reflection spectrum
Sensitivity to temperature changes	High	Average
Sensitivity to the surrounding RI	Very high	Low

**Table 6:** Examples of fiber optic sensors based on TOF structures with additional materials.

Type	Active Material	Principle/Method	Measured Factor	Range	Sensitivity	Ref.
TOF	Au NPs	LSPR	BSA	1 ng/mL –10 mg/mL	19.46 nm/log(ng/mL)	[90]
TOF	Au film	SPR	Salinity	0–40‰	0.708 nm/‰	[84]
Twisted TPOF *	-	Coupling method	Alcohol: ethanol, propanol, butanol, and pentanol	-	506 %/RIU	[209]
U-shaped TPOF	Au film	SPR	RI	1.335–1.41	1534.53 nm/RIU	[32]
TOF	Au and PVA	SPR	Humidity Temperature	46–93 %RH	1.542 nm/%RH	[89]
TOF	Pd	SPR	Hydrogen	0.125–2.00 % H_2_	−18.645 %	[88]
Cascaded SMTF **	Protein antibodies	Cascaded interferometric effect	Dengue II E and SARS-CoV-2 S proteins	0.0–1.0 nM	6.91 nm/nM and 9.96 nm/nM	[210]
TOF	PVA/DE	EW	Paper moisture content	-	0.1662 %/%ω	[122]
TOF	MoS_2_ and PDMS	Interferometer	Bending curvature Temperature	4.31–6.10 m^−1^ 35–42 °C	23.62 nm/m^−1^ 2.70 nm/m^−1^ 1.10 nm/°C	[124]
TOF sandwiched in PDMS film	PDMS with NHSA ***	Self-mixing interference	Blood pressure	-	0.0138 mV/kPa	[126]
TOF modified using femtosecond laser pulses	PDMS	Mach–Zehnder interferometer	Temperature	30–65 °C	616.3 pm/°C	[125]
TOF	GO–Fe_3_O_4_	EW	Magnetic field	0–60 mT	99–324 pW/mT	[140]
2-twisted TOF	-	SPP, multi-mode interference	Temperature Tensile strain	30–60 °C 0–90 με	−73.7 pm/°C −163.5 pm/με	[141]
2-twisted TOF	Graphene				−127.2 pm/°C −173.9 pm/με	
3-twisted TOF					−66.2 pm/°C −173.4 pm/με	[142]
4-twisted TOF					−61.7 pm/°C −103.2 pm/με	
Non-adiabatic TOF	- GO/PVA	EW	Humidity	20–99.9 %	0.00106 a.u. (%)^−1^ 0.00624 a.u.(%)^−1^	[139]
TOF	PDMS and GO	EW	MUC1 protein	10–400 μg/ml	2.11 dB/log(C(μg/mL)) 0.11 pM (detection limit)	[137]
TIT	ZnO-NPs	EW	Creatine Ethanol solution	0–2000 μM 0–100%	0.11 a.u./μM 4.06 a.u./%	[106]
S-tapered	SiO_2_ NPs	EW	Humidity	83.8–95.2 %RH	1.1718 nm/%RH and 0.441 dB/%RH	[108]
TIT	AuNPs/MoS_2_NP/CeO2-Ns	LSPR	Alanine aminotransferase	10–1000 U/L	4.1 pm/(U/L)	[107]
TOF	Aldehyde modifier	EW	Leptospira DNA	0.1–1.0 nM	1.2862 nm/nM	[112]
TOF	Zeolite imidazole framework ZIF-8	The Vernier effect	Ethanol (gas concentration) RI	0–140 ppm -	0.1411 nm/ppm 18,366.17 nm/RIU	[111]
TOF	PVA	EW	Humidity	30–90 %RH	0.119 nm/%RH	[130]
Asymmetric TFBG	-	Fresnel reflection	Temperature	30–350 °C	12.3 pm/°C	[196]
FBG	-	Fresnel reflection	Temperature	0–140 °C	717 1/°C	[197]
SCF FBG	-	Fresnel reflection	Vector bending	-	127 pm/m^−1^	[199]
Chirped TFBG	-	Fresnel reflection/Fabry–Perot	Strain Temperature	-	5 μm/με 8.9 Chirped 6 pm/°C	[200]
Etched chirped TFBG tip	-	Fabry–Perot cavity	Temperature	25–65 °C	~11 pm/°C	[36]
MKR	PDMS		Acoustic sensor		−135.8 dB	[154]
MKR	PDMS		Strain sensor		94.5 pm/N	[152]
DMKR	-	The Vernier effect	RI and temperature	1.3375–1.3359 0–25.3 °C	12523 nm/RIU 0.91998 nm/°C	[150]
MKR–U-shaped MZ interferometer	Agarose	The Vernier effect	Humidity	60–95% RH	2.442 nm/%RH	[158]

* tapered POF; ** single-mode tapered fiber; *** nanohemispherical array.

## Data Availability

No new data were created or analyzed in this study.
